# Supplementary material for: First report of MDR virulent Pseudomonas aeruginosa in apparently healthy Japanese quail (Coturnix japonica) in Bangladesh
Source: PLoS One. 2025 Jan 24;20(1):e0316667. doi: 10.1371/journal.pone.0316667 (PMC11761672; doi:10.1371/journal.pone.0316667)
Supplement: S1 Table — (DOCX) [file pone.0316667.s001.docx]

**Supplementary information: S1 Table**

***PSEUDOMONAS AERUGINOSA* FROM JAPANESE QUAIL**

**First Report of MDR Virulent *Pseudomonas aeruginosa* in Apparently Healthy Japanese Quail (*Coturnix japonica*) in Bangladesh**

^ǂ^Alamgir Hasan, ^ǂ^Md Tanjir Ahmmed, Bushra Benta Rahman Prapti, Aminur Rahman, Tasnim Islam, Chandra Shaker Chouhan, A. K. M. Anisur Rahman, and Mahbubul Pratik Siddique*

*Department of Microbiology and Hygiene, ^†^Department of Medicine, Bangladesh Agricultural University, Mymensingh-2202

^1^Corresponding author: [mpsiddique@bau.edu.bd](mailto:mpsiddique@bau.edu.bd)

^ǂ^ these authors equally contributed

**Table S1.** Gender wise positive distribution of *P. aeruginosa* at different areas

| **Area of sampling** | **Isolate ID** | **Male** | **Female** |
| --- | --- | --- | --- |
| Mymensingh Sadar | PM1 | Rectum |  |
|  | PM2 |  | Oral |
|  | PM3 |  | Oral |
|  | PM4 |  | Rectum |
|  | PM5 | Oral+Rectum |  |
|  | PM6 |  | Rectal+Oral |
|  | PM7 |  | Oral |
|  | PM8 |  | Rectum |
|  | PM9 |  | Oral |
|  | PM10 |  | Rectum |
|  | PM11 |  | Rectum |
| Jamalpur | PM12 |  | Oral |
|  | PM13 | Rectum |  |
|  | PM14 | Rectum |  |
|  | PM15 |  | Rectum |
|  | PM16 |  | Rectum |
| Narsingdi | PM17 | Oral |  |
|  | PD18 |  | Rectum |
|  | PD19 | Rectum |  |
|  | PD20 |  | Oral+Rectum |
|  | PD21 | Rectum |  |
|  | PD22 | Rectum |  |
|  | PD23 |  | Rectum |
| Gazipur | PD24 |  | Rectum |
|  | PD25 | Rectum |  |
| **Total positive** | | **9 (36%)** | **16 (64%)** |
