# Supplementary material for: First report of MDR virulent Pseudomonas aeruginosa in apparently healthy Japanese quail (Coturnix japonica) in Bangladesh
Source: PLoS One. 2025 Jan 24;20(1):e0316667. doi: 10.1371/journal.pone.0316667 (PMC11761672; doi:10.1371/journal.pone.0316667)
Supplement: S2 Table — (DOCX) [file pone.0316667.s002.docx]

**Supplementary information: S2 Table**

***PSEUDOMONAS AERUGINOSA* FROM JAPANESE QUAIL**

**First Report of MDR Virulent *Pseudomonas aeruginosa* in Apparently Healthy Japanese Quail (*Coturnix japonica*) in Bangladesh**

^ǂ^Alamgir Hasan, ^ǂ^Md Tanjir Ahmmed, Bushra Benta Rahman Prapti, Aminur Rahman, Tasnim Islam, Chandra Shaker Chouhan, A. K. M. Anisur Rahman, and Mahbubul Pratik Siddique*

*Department of Microbiology and Hygiene, ^†^Department of Medicine, Bangladesh Agricultural University, Mymensingh-2202

^1^Corresponding author: [mpsiddique@bau.edu.bd](mailto:mpsiddique@bau.edu.bd)

^ǂ^ these authors equally contributed

**Table S2.** Sample wise positive distribution of different virulence genes of *P. aeruginosa*

| **Isolates ID** | ***exo*A** | ***exo*S** | ***exo*T** | ***exo*U** | ***exo*Y** | ***rhl*AB** | ***rhl*R** | ***rh*lI** | ***las*I** | ***las*A** |
| --- | --- | --- | --- | --- | --- | --- | --- | --- | --- | --- |
| PM1 | P | P | P | N | P | P | P | P | N | N |
| PM2 | P | P | P | N | P | N | P | P | N | N |
| PM3 | P | P | N | N | N | P | P | P | N | N |
| PM4 | P | P | P | N | P | P | P | P | N | N |
| PM5 | P | P | N | N | P | P | P | P | N | N |
| PM6 | P | P | P | N | P | N | P | N | N | N |
| PM7 | P | P | P | N | P | P | P | P | N | N |
| PM8 | P | P | N | N | P | P | P | N | N | N |
| PM9 | P | P | N | N | N | P | P | N | N | N |
| PM10 | P | N | P | N | P | P | P | P | N | N |
| PM11 | P | P | P | N | P | N | P | N | N | N |
| PM12 | P | P | N | N | P | P | P | N | N | N |
| PM13 | P | P | P | N | P | P | P | P | N | N |
| PM14 | P | P | P | N | P | P | P | N | N | N |
| PM15 | P | P | P | N | P | P | P | P | N | N |
| PM16 | P | P | P | N | P | P | P | P | N | N |
| PM17 | P | P | N | N | P | P | P | P | N | N |
| PD18 | P | P | P | N | P | P | P | P | N | N |
| PD19 | P | N | P | N | N | P | P | P | N | N |
| PD20 | P | P | P | N | P | N | P | N | N | N |
| PD21 | P | P | P | N | P | P | P | N | N | N |
| PD22 | P | P | N | N | P | P | P | P | N | N |
| PD23 | P | N | N | N | N | N | P | N | N | N |
| PD24 | P | P | P | N | P | P | P | P | N | N |
| PD25 | P | P | N | N | P | N | P | P | N | N |

**Note:** Here P= Pisitive, N = Negative
