# Supplementary material for: First report of MDR virulent Pseudomonas aeruginosa in apparently healthy Japanese quail (Coturnix japonica) in Bangladesh
Source: PLoS One. 2025 Jan 24;20(1):e0316667. doi: 10.1371/journal.pone.0316667 (PMC11761672; doi:10.1371/journal.pone.0316667)
Supplement: Attachment — Submitted filename: Response to Edditors and Reviewers.docx [file pone.0316667.s004.docx]

**Edditor**

**Comments to authors and RESPONSE**

**Manuscript ID:** PONE-D-24-41634

**Title:** First Time Detail Virulence and antibiogram profiling of *Pseudomonas aeruginosa* isolated from apparently healthy Japanese quail (*Coturnix japonica*) in Bangladesh

**Modified title:** **First Report of MDR Virulent *Pseudomonas aeruginosa* in Apparently Healthy Japanese Quail (*Coturnix japonica*) in Bangladesh**

**Response**: We are thankful to the editor and reviewers for their valuable and constructive comments, which helped us improve the quality of our manuscript. We have carefully revised the manuscript considering their suggestions and believe that the revised version meets the expectations of the editor and reviewers. Our responses to the reviewers’ specific comments are provided in red coloured text in the revised manuscript.

Journal Requirements:

When submitting your revision, we need you to address these additional requirements.

3. We note that your Data Availability Statement is currently as follows: [All relevant data are within the manuscript and its Supporting Information files]

Please confirm at this time whether or not your submission contains all raw data required to replicate the results of your study. Authors must share the “minimal data set” for their submission. PLOS defines the minimal data set to consist of the data required to replicate all study findings reported in the article, as well as related metadata and methods (<https://journals.plos.org/plosone/s/data-availability#loc-minimal-data-set-definition>).

For example, authors should submit the following data:

- The values behind the means, standard deviations and other measures reported;

- The values used to build graphs;

- The points extracted from images for analysis.

Authors do not need to submit their entire data set if only a portion of the data was used in the reported study.

If your submission does not contain these data, please either upload them as Supporting Information files or deposit them to a stable, public repository and provide us with the relevant URLs, DOIs, or accession numbers. For a list of recommended repositories, please see <https://journals.plos.org/plosone/s/recommended-repositories>.

If there are ethical or legal restrictions on sharing a de-identified data set, please explain them in detail (e.g., data contain potentially sensitive information, data are owned by a third-party organization, etc.) and who has imposed them (e.g., an ethics committee). Please also provide contact information for a data access committee, ethics committee, or other institutional body to which data requests may be sent. If data are owned by a third party, please indicate how others may request data access.

**Response:** The data generated during this study is included in the manuscript and its associated supplementary files.

4. PLOS ONE now requires that authors provide the original uncropped and unadjusted images underlying all blot or gel results reported in a submission’s figures or Supporting Information files.

**Response:** We thank to the editor for this comment. The raw blot/gel images are included in the Figure section not to Supporting Information.

5. We note that you have included the phrase “data not shown” in your manuscript. Unfortunately, this does not meet our data sharing requirements. PLOS does not permit references to inaccessible data. We require that authors provide all relevant data within the paper, Supporting Information files, or in an acceptable, public repository. Please add a citation to support this phrase or upload the data that corresponds with these findings to a stable repository (such as Figshare or Dryad) and provide and URLs, DOIs, or accession numbers that may be used to access these data. Or, if the data are not a core part of the research being presented in your study, we ask that you remove the phrase that refers to these data.

**Response:** We thank to the editor for this comment. Sorry for such inconvenience and we have removed **“data not shown”** from the manuscript accordingly.

6. We note that [Figure 1] in your submission contain [map/satellite] images which may be copyrighted. All PLOS content is published under the Creative Commons Attribution License (CC BY 4.0), which means that the manuscript, images, and Supporting Information files will be freely available online, and any third party is permitted to access, download, copy, distribute, and use these materials in any way, even commercially, with proper attribution. For these reasons, we cannot publish previously copyrighted maps or satellite images created using proprietary data, such as Google software (Google Maps, Street View, and Earth). For more information, see our copyright guidelines: <http://journals.plos.org/plosone/s/licenses-and-copyright>.

**Response:** Thanks to the editor for this comment. Now, we have produced Figure 2 in Python using Matplotlib and Geopandas libraries. We have downloaded the district level Bangladesh shape file from GADM (https://gadm.org/)

**Reviewer #1:**

**Comments to authors and RESPONSE**

-The current study is interesting; however, the authors should address the following comments to improve the quality of the manuscript:

-The manuscript should be revised for English editing and grammar mistakes.

**Response:** Thanks to the reviewer for this comment. We have critically checked the manuscript for English editing and fixing grammar mistakes.

-Please write the scientific names of bacterial pathogens and genes in the correct form all over the manuscript and the references section.

**Response:** We thank the reviewer for this comment. We have italicized the scientific names of bacterial pathogens and genes throughout the manuscript and in the reference section.

Title:
I think the work would benefit from the title that contains the main conclusion of the study (should be derived from the conclusion). Please modify the title.

**Response:** We have modified the title as “**First Report of MDR Virulent *Pseudomonas aeruginosa* in Apparently Healthy Japanese Quail (*Coturnix japonica*) in Bangladesh**”.

Abstract:
- The abstract must illustrate the used methods and the most prevalent results (give more hints about methods and results). Besides, rephrase the aim of the work and the main conclusion of your findings.

**Response:** We have modified the abstract according to the suggestions of the reviewer.

- Add the full expression before the abbreviations.

**Response:** Thank you. We have elaborated before using abbreviated form.

-Introduction: (it needs to be more informative):

-Give a hint about the virulence factors and the mechanism of disease occurrence, and infections caused by *P. aeruginosa.*

**Response:** According to the reviewer’s comments, we have added/updated the virulence factors, the mechanism of disease occurrence and infections caused by *P. aeruginosa.*

-Illustrate the mechanism of action different virulence factors of P. aeruginosa.

**Response:** The mechanism of action of different virulence factors had already been described in the submitted manuscript.

-Rephrase the aim of the work to be clear and better sound.

**Response:** The aims of the works have been rephrased to improve readability and clarity.

Material and methods:

-Support all methods with updated specific references.

**Response:** All methods have been cited with appropriate references.

•Add the company, city, and country of the used chemicals and reagents.

**Response:** The company, city and country of the used chemicals and reagents have been added.

-Isolation and identification:

Discuss in detail the methods of isolation and identification of P. aeruginosa. Besides, specific references should be added.

**Response:** Modified according to the suggestions of the reviewer.

•Add the company, city, and country of the used bacterial media and reagents that were used in the biochemical identification of isolates. Also, enumerate all used biochemical reactions.

**Response:** The company, city and country of the used bacterial media and reagents have been added.

-PCR based detection of other antimicrobial resistance genes should be performed. Afterwards, the correlation between phenotypic and genotypic multidrug resistance should be performed.

**Response:** In our study, we did not detect antimicrobial resistance genes among the *Pseudomonas aeruginosa* isolates. However, we did identify virulence genes and conducted a correlation analysis between the phenotypic resistance profiles and the virulence characteristics of the isolates. Please see Table 5 and Figure 7.

-MLST should be carried out to illustrate the genetic relatedness among the retrieved isolates (If available).

**Response:** We appreciate the insightful suggestion from the reviewer regarding the use of MLST to assess genetic relatedness between the recovered isolates. While we acknowledge the value of MLST in providing detailed insights into bacterial population structures, unfortunately, due to resource constraints, we were unable to conduct MLST analysis in this study. Our primary focus was on investigating the prevalence, risk factors, virulence, and antimicrobial resistance profiles of *P. aeruginosa* in apparently healthy quail birds. We believe that the comprehensive data presented in our study, encompassing both phenotypic and genotypic aspects, contributes significantly to understanding the potential public health implications associated with *P. aeruginosa* in quail populations.

-Results:

-Please add a starting paragraph to the results section to briefly introduce the topic, your goals and hypothesis and a short summary of what you did in this work.

**Response:** We have added a starting paragraph to briefly introducing the topic, our goal, hypothesis and a short summary of what have done.

-Antimicrobial susceptibility testing:

• Illustrate in a new table the occurrence of MDR (Multidrug resistance) among the recovered isolates as the following (illustrate the names of the antimicrobial classes and different antibiotics):

No. of strains%Type of resistance R, MDR, and XDRPhenotypic multidrug resistance
(Antimicrobial classes and different antibiotics). The antibiotic-resistance genes

-Tables 6 and 7: Address the multidrug resistance patterns in the tables. I couldn't find it (MDR, XDR, and PDR)

**Response:** Cordial thank and gratitude again to the honourable reviewer for this kind comments and useful suggestion. We have added a new table (Table 5 in the revised version) illustrating the details phenotypic resistance and virulence profiling, indicating their MARI, MDR, XDR, PDR, their percent prevalence. Due to repetition of same theme, we have deleted Table 5 of previous version.

MLST should be carried out to illustrate the genetic relatedness among the retrieved isolates.

**Response:** Thank you again. We already added the explanation in the Materials and Methods section of the Reviewer’s comments. We should explore and incorporate this striking feature in our future researches, if resources are available.

-Support your findings with more illustrating figures. Besides, increase the resolution of all figures (must be 600 dpi).

**Response:** We have improved the resolution of all figures (at least 600 dpi)

**Moreover, for more clarification, even we changed figure 2, 3, 5, and 6 with new look and higher resolution**

-Discussion:

-The authors are advised to illustrate the real impact of their findings without repetition of results.

**Response:** We have discussed the real impact of our findings without repeating the results.

-Please illustrate the role of virulence genes and virulence determinants of P. aeruginosa in the pathogenesis of the disease.

**Response:** We have discussed different mechanisms of antibacterial resistance in *P. aeruginosa*.

-Please illustrate different mechanisms of antibacterial resistance P. aeruginosa.

**Response:** We have discussed different mechanisms of antibacterial resistance in *P. aeruginosa*.

-Illustrate the essential role of biofilm in the occurrence of infection.

**Response:** We have discussed different mechanisms of antibacterial resistance in *P. aeruginosa.*

-Conclusion
-Should be rephrased to be sounded. A real conclusion should focus on the question or claim you articulated in your study, which resolution has been the main objective of your paper?

**Response:** We have rewritten the conclusion as suggested.

**Reviewer #2:**

**Comments to authors and RESPONSE**

: Dear Editor:-

I think this article is very good regarding the originality of the idea at which the field of study is so interesting and medically important.

The topic of manuscript is timely and will be of interest to the readers of the journal. Also, the manuscript is very well written and the ideas flow logically.
Dear authors you are doing well and that’s look great work. A few issues, however, need to be addressed;

In line 13 page 2 Abstract: Abstract was too long, must be 250 words.

**Response:** We thank the reviewer for this comment. The abstract has been revised and shortened to 300 words in accordance with the journal guidelines.

In line 13 page 2 Give a brief explanation of the significance of Pseudomonas aeruginosa for bird health and its effects on the poultry trade. This might facilitate the setting of your research.

**Response:** We thank the reviewer for this comment. The significance of *Pseudomonas aeruginosa* for bird health and its impact on the poultry industry has been incorporated into the abstract, as mentioned in the previous comment.

In line 17 page 2 Provide details on how the quail were selected for sampling. Also, mention whether the birds were healthy or showing signs of illness.

**Response:** The abstract has been revised in accordance with the reviewer’s suggestions, as mentioned in the previous comment.

What effects do the virulence genes (*exoA*, *rhlR*, etc.) that have been found have on *P. aeruginosa's* pathogenicity in quail? Could you explain more about the relevance of other genes' varied distribution.?

**Response:** We thank the reviewer for this comment. The presence of virulence genes such as *exo*A, *rhl*R, *exo*S, *exo*Y, and *rhl*I encodes toxins, enzymes, and various secretory proteins that facilitate bacterial persistence, colonization, survival, and infection of the host under adverse conditions. These genes contribute to biofilm formation, induce quorum sensing, promote cell death, cause tissue damage, and manipulate host cellular processes. The specific roles of these virulence genes have been incorporated into the introduction section as suggested.

Is there a biological or environmental reason why female quail are more likely than male quail to host *Pseudomonas* spp.?

**Response:** We thank the reviewer for this comment. Hypothetically, we propose that female quail may be more susceptible to certain *Pseudomonas* species due to hormonal influences, differences in immune responses, and behavioral patterns. Additionally, genetic predispositions, including differences in gene expression related to immune function, could play a role in increasing susceptibility in females compared to males. These factors may explain why female quail are more likely to host *Pseudomonas* spp. than their male counterparts.

In line 28 page2 What biological or environmental factors could explain the increased odds of female quail harboring *Pseudomonas* spp. compared to males?

**Response:** We thank the reviewer for this comment. Several factors may contribute to the increased odds of female quail harboring *Pseudomonas* spp. compared to males. From a biological perspective, female quail secrete sex hormones such as estrogen and progesterone, which can influence immune function and potentially enhance susceptibility to infections by modulating immune responses. Additionally, differences in the gut and skin microbiomes between females and males may create conditions more favorable for bacterial colonization in females. From an environmental perspective, female quail might be more frequently exposed to environments with a higher prevalence of *Pseudomonas* spp., leading to increased contact with contaminated substrates. These combined factors could explain the observed difference in susceptibility.

In line 35 page 2 Given that exoU, lasI, and lasA were not detected in any isolates, how might this influence the virulence potential of these strains?

**Response:** We thank the reviewer for this comment. We used approximately 10 primer sets to investigate the association with virulence, where all isolates tested positive for 7 virulence genes, while the remaining 3 were absent. Based on these findings, we conclude that the isolates exhibit virulence on a majority basis. The corresponding statement has been corrected and updated in the revised abstract.

In line 84 page 5 Regarding *P. aeruginosa* from quails, how does the rise in multidrug resistance (MDR) correspond to developments in resistance in human medicine?

**Response:** Thanks to the reviewer for this valuable comment. The co-relation of MDR in between livestock animal and human health has been added in the introduction section.

In line 88 page 5 To enhance introduction section add the following reference: Rawaa A. Hussein, Shaymaa H. AL-Kubaisy, Mushtak T.S. Al-Ouqaili. The influence of efflux pump, outer membrane permeability and β-lactamase production on the resistance profile of multi, extensively and pandrug resistant Klebsiella pneumoniae. Journal of Infection and Public Health, Volume 17, Issue 11, 2024. <https://doi.org/10.1016/j.jiph.2024.102544>.

**Response:** This reference has already been added in the introduction section.

In line 90 page 5 Why are quail farms mentioned in this particular setting? Could you explain on how common AMR is, particularly in quail farms as opposed to other livestock?

**Response:** We thank the reviewer for this comment. The common relationship between the occurrence of antimicrobial resistance (AMR) in livestock animals and its impact on human health has already been incorporated into the introduction section, as mentioned in the previous comment.

In line 100 page 6 To enhance introduction section add the following reference: Alduhaidhawi AHM, AlHuchaimi SN, Al-Mayah TA, Al-Ouqaili MTS, Alkafaas SS, Muthupandian S, Saki M. Prevalence of CRISPR-Cas Systems and Their Possible Association with Antibiotic Resistance in Enterococcus faecalis and Enterococcus faecium Collected from Hospital Wastewater. Infect Drug Resist. 2022 Mar 19;15:1143-1154. doi: 10.2147/IDR.S358248. PMID: 35340673; PMCID: PMC8942119.

**Response:** The reference mentioned has already been included in the introduction section.

In line 118 page 7 What guidelines were applied when choosing the particular areas for the sample—Jamalpur, Mymensingh, Gazipur, and Narsingdi? Do these regions reflect the larger quail farming sector in Bangladesh?

**Response:** We thank the reviewer for this comment. The sampling areas were selected based on the availability of birds.

In line 119 page 7 Why were oral and cloacal swabs the only ones used to get samples? Would other sample types—like environmental or fecal samples—offer more thorough insights?

**Response:** We thank the reviewer for this comment. For sample collection from live birds, oral and cloacal swabs are considered the ideal sources for isolating *P. aeruginosa*. Fecal samples, on the other hand, are more prone to contamination due to exposure to environmental settings. To avoid this risk, we collected samples from the cloaca. Regarding environmental samples, we agree with the reviewer and will consider collecting such samples in future studies.

In line 122 page 7 Why are samples from male and female quails being collected in equal numbers, and why specifically? Regarding illness susceptibility or AMR prevalence, were gender variations taken into account?

**Response:** Thanks to the reviewer for this valuable comment. We have collected both male and female sample equally to compare the prevalence of AMR and virulence gene distribution between male and female birds.

In line 149 page 8 On one hand authors worked with boiling and thawing for DNA extraction technique, but why Ethidium bromide was used. Ethidium bromide- a carcinogen!

**Response:** We agree with the suggestion and will use Safe DNA stain in the future. Unfortunately, we did not have access to Safe DNA stain at the time of this experiment.

In line 153 page 8 What is the clinical significance of detecting the specified virulence-associated genes in your study? How does the presence of these genes correlate with pathogenicity or virulence in Pseudomonas?

**Response:** The presence of virulence genes indicates the production of toxins, enzymes, and various secretory proteins that enable the bacteria to persist, colonize, survive, and infect the host under adverse conditions. Therefore, we can infer that if virulence genes are present, the bacteria are likely to be pathogenic

In line 232 page 14 In what ways was turbidity assessed or quantified in your cultures? When evaluating the turbidity levels in LB broth, nutritional broth, and BHI broth, did you employ any particular methods or tools?

**Response:** Thanks to the reviewer for this important comment. Sorry for avoiding this point. The particular method for measuring turbidity has been added in the Material and Method mentioned below:

In line 267 page 16 Results by sample type and gender are shown in Table 2. Why are the prevalence statistics for male and female birds presented differently, in your opinion? What knowledge does this analysis of gender differences offer?

**Response:** We have collected samples from four different districts, including both male and female birds, to determine the distribution of pathogenicity among them. To present this data clearly, we have arranged the Table 2, which provides an overview of the pathogenicity distribution. Additionally, the table suggests that farmers should pay extra attention to female birds during husbandry.

In line 273 page 16 What underlying variables could be responsible for the higher probability of Pseudomonas spp. being present in quail from Narsingdi as opposed to Mymensingh Sadar?

**Response:** Thanks to the reviewer for this comment. The bird farm in Mymensingh district is in the city area, which benefits from a good communication system, access to veterinary services, the presence of a university, vaccines, and other support. In contrast, the bird farm in Narsingdi is situated in rural areas, where there is a lack of veterinary facilities and limited knowledge about poultry diseases among bird handlers. This difference in infrastructure and resources may explain why the incidence of infection caused by Pseudomonas spp. was higher in quail from Narsingdi compared to Mymensingh.

In line 290 page 17 The absence of genes such as *exo*U, *las*I, and *las*A is noteworthy. What implications does this have for the virulence potential of these isolates? Could their absence indicate a particular environmental adaptation?

**Response:** Thanks to the reviewer for this comment. The *exo*U, *las*I, and *las*A are associated with the production of cytotoxin, regulation of quorum-sensing system, and secretion of protease enzyme, all of which contribute to increased virulence in *Pseudomonas aeruginosa.* However, the absence of these virulence genes does not necessarily rule out pathogenicity, as other virulence factors may compensate for their absence, or the bacteria may adopt chronic infection strategies. Additionally, the lack of these genes may indicate an adaptation to environments where tissue invasion is less critical, or where alternative survival strategies, such as biofilm formation, are employed to enhance resilience against host defenses and antibiotics.

In line 293 page 17 Has any genetic diversity study been done on the 25 isolates of P. aeruginosa? In what ways could genetic variation affect the way virulence genes are distributed?

**Response:** Thanks to the reviewer for this comment. Several methods, such as Whole Genome Sequencing, Multi-Locus Sequence Typing, Phylogenetic Analysis, and PCR amplification of virulent genes, can be used to detect the genetic diversity of bacteria. In our study, we performed PCR amplification of virulent genes for 25 *Pseudomonas aeruginosa* isolates. Additionally, one representative isolate from the 25 *P. aeruginosa* isolates was selected for Whole Genome Sequencing, as described in the results section.

In line 308 page 19 It is remarkable that 72% of specimens have intermediate streptomycin resistance. In the setting of treatment, how should this be understood, and what kind of follow-up testing is recommended?

**Response:** Thanks to the reviewer for this comment. The intermediate resistance of certain drugs suggests that some bacterial isolates may still be susceptible to the drug at higher concentrations. However, to better understand this phenomenon, alternative antibiotics with more reliable efficacy against resistant strains may be more suitable. For follow-up testing, Antimicrobial Susceptibility Testing (AST), including the determination of Minimum Inhibitory Concentration (MIC), is essential. MIC helps identify the lowest concentration of an antibiotic needed to inhibit bacterial growth. Additionally, antibiogram profiling, which tests bacterial isolates against various antibiotics to map out resistance patterns, can provide valuable insights. Regular clinical assessments and laboratory tests, such as blood or urine cultures, should be conducted to monitor treatment effectiveness and adjust as needed. Monitoring resistance patterns in bacterial populations is also crucial to ensure that treatment strategies remain aligned with current trends in resistance.

In line 326n page 20 Nine of the 18 antibiotics evaluated in the study demonstrated susceptibility. How did these antibiotics come to be chosen? Were they chosen because they were commonly used antibiotics in clinical settings, or because they were relevant to veterinary medicine?

**Response:** Thanks to the reviewer for this comment. We have selected the antibiotics based on the Clinical and Laboratory Standards Institute (CLSI, 2019) guidelines, as outlined in the methods section.

In line 368 page 23 Could you provide more details about how your research on quail birds in particular advances our knowledge of P. aeruginosa's potential harm to public health? In what way does this study contribute to the overall understanding of P. aeruginosa epidemiology?

**Response:** Thanks to the reviewer for this comment. Additional information about how this research on quail birds specifically contributes to our understanding of *Pseudomonas aeruginosa*'s potential harm to public health has been included in the discussion section.

In line 326 page 24 To enhance discussion section add the following reference: Murshid RM, Al-Ouqaili MTS, Kanaan BAJ. Microbial Vaginosis and its Relation to Single or Multi-Species Biofilm in Iraqi Women: Clinical and Microbiological Study. Pak J Biol Sci. 2024 Jul;27(8):404-412. doi: 10.3923/pjbs.2024.404.412. PMID: 39300677.

**Response:** Thanks to the reviewer for this comment. To enhance discussion the suggested reference has been added to the discussion section.

In line 388 page 24 According to result of this research, the likelihood of contracting P. aeruginosa is six times higher in young birds (less than 8 weeks old). In younger birds, what particular biochemical or immunological pathways could explain this heightened vulnerability? Has the literature put forth or examined any plausible theories?

**Response:** Thanks to the reviewer for this valuable comment. The young birds are more vulnerable to *Pseudomonas aeruginosa* due to several factors. Their immune system is still immature, which prevents the full development of specific antibodies and T cells, and results in a lower number of phagocytic cells during the early weeks of life. This leads to poor recognition and elimination of pathogens like *P. aeruginosa*. Additionally, young birds often face nutritional deficiencies and have immature mucosal surfaces, which increase the permeability and susceptibility to infections, allowing pathogens like *P. aeruginosa* to colonize more easily. This phenomenon has been widely discussed in numerous reputable journals. An explanation of the increased infectivity of young birds has been added to the discussion section.

In line 401page 24 To enhance discussion section add the following reference: •Saleh RO, Al-Ouqaili MTS, Ali E, Alhajlah S, Kareem AH, Shakir MN, Alasheqi MQ, Mustafa YF, Alawadi A, Alsaalamy A. lncRNA-microRNA axis in cancer drug resistance: particular focus on signaling pathways. Med Oncol. 2024 Jan 9;41(2):52. <http://doi.org/> 10.1007/s12032-023-02263-8.

**Response:** Thanks to the reviewer for this comment. To enhance discussion, the suggested reference has been added to the discussion section.

In line 450 page 27 Conclusion should be objective with further perspective or should add at least a few sentences about future study/future perspective of it.

**Response:** Thanks to the reviewer for this comment. The future plane of this research has been added to the conclusion section.
