# Supplementary material for: First report of MDR virulent Pseudomonas aeruginosa in apparently healthy Japanese quail (Coturnix japonica) in Bangladesh
Source: PLoS One. 2025 Jan 24;20(1):e0316667. doi: 10.1371/journal.pone.0316667 (PMC11761672; doi:10.1371/journal.pone.0316667)
Supplement: Attachment — Submitted filename: Reviewer comments-PONE-D-24-41634.pdf [file pone.0316667.s003.pdf]

PLOS ONE

First Time Detail Virulence and antibiogram profiling of Pseudomonas aeruginosa isolated from apparently healthy Japanese quail (Coturnix japonica) in Bangladesh

--Manuscript Draft--

**Manuscript Number:**	PONE-D-24-41634
**Article Type:**	Research Article
**Full Title:**	First Time Detail Virulence and antibiogram profiling of Pseudomonas aeruginosa isolated from apparently healthy Japanese quail (Coturnix japonica) in Bangladesh
**Short Title:**	Virulence and antibiogram profiling of Pseudomonas aeruginosa from Japanese quail (Coturnix japonica)
**Corresponding Author:**	Mahbubul Pratik Siddique, Ph.D.<br>Bangladesh Agricultural University<br>Mymensingh, Mymensingh BANGLADESH
**Keywords:**	Swab, Isolation, Risk factors, MD, Antibiogram, PCR
**Abstract:**	Pseudomonas aeruginosa (P. aeruginosa) is the predominant species associated with global poultry infections, particularly affecting Japanese quail (Coturnix japonica), characterized by its elevated transmissibility and mortality across all age groups. The objectives of this study were to isolate and identify P. aeruginosa from Japanese quails, identify risk factors, detect virulence genes and antibiotic resistance. Sampling oral and rectal swabs from 110 quail birds) across Mymensingh, Jamalpur, Gazipur and Narsingdi districts (n=110x2=220, we used cultural, morphological, biochemical, and polymerase chain reaction (PCR) methods for bacterial isolation and identification. Antibiotic sensitivity was assessed through disc diffusion against 18 commonly used antibiotics, and virulence genes were detected through PCR. We used multivariable logistic regression analysis to identify risk factors for P. aeruginosa infection. Both conventional and PCR methods revealed 25 (22.73%) quail birds as positive for P. aeruginosa. In Narsingdi, quail birds were found to be five times more likely to harbour Pseudomonas spp. (OR: 5.02; 95% CI:1.34–18.84) compared to those in Mymensingh Sadar. Quail birds younger than eight weeks had nearly six times higher odds (OR: 5.93; 95% CI: 1.96-17.91) of carrying Pseudomonas spp. compared to their older counterparts. Similarly, female quail birds showed almost four times higher odds of harbouring Pseudomonas spp. (OR: 3.77; 95% CI: 1.30-10.93) than male birds. All 25 P. aeruginosa isolates exhibited multi drug-resistance (MDR) pathogen, showing resistance to Aztreonam, Doxycycline, Chloramphenicol, and Florfenicol, yet maintaining susceptibility to Meropenem, Ceftriaxone, Cephradine, Streptomycin, Neomycin, Gentamycin, Cotrimoxazole, Ciprofloxacin, and Levofloxacin. Virulence gene distribution revealed consistent presence of exoA and rhlR in all isolates, while exoS, exoY, rhlI, rhlAB, and rhlR showed variable distribution. No isolate tested positive for exoU, lasI and lasA genes. Our findings highlight the prevalence of multi-drug-resistant and virulent P. aeruginosa in apparently healthy quail birds, reared in Mymensingh and Dhaka divisions of Bangladesh. This emphasizes the urgent need for a comprehensive 'One Health' approach to proactively address and mitigate the potential for future disasters caused by this organism in quail and human populations.
**Order of Authors:**	Alamgir Hasan, M.S.
	Md Tanjir Ahmmed, M.S.
	Bushra Benta Rahman Prapti, M.S.
	Aminur Rahman, M.S.
	Tasnim Islam, M.S.
	Chandra Shaker Chouhan, M.S.
	A. K. M. Anisur Rahman, Ph.D.
	Mahbubul Pratik Siddique, Ph.D.
**Additional Information:**	

Powered by Editorial Manager® and ProduXion Manager® from Aries Systems Corporation

Question	Response
**Financial Disclosure**<br><br>Enter a financial disclosure statement that describes the sources of funding for the work included in this submission. Review the submission guidelines for detailed requirements. View published research articles from *PLOS ONE* for specific examples.<br><br>This statement is required for submission and **will appear in the published article** if the submission is accepted. Please make sure it is accurate.<br><br>**Funded studies**<br>Enter a statement with the following details:<br>• Initials of the authors who received each award<br>• Grant numbers awarded to each author<br>• The full name of each funder<br>• URL of each funder website<br>• Did the sponsors or funders play any role in the study design, data collection and analysis, decision to publish, or preparation of the manuscript?<br><br>Did you receive funding for this work?	The author(s) received no specific funding for this work.
**Competing Interests**<br><br>Use the instructions below to enter a competing interest statement for this submission. On behalf of all authors, disclose any competing interests that could be perceived to bias this work—acknowledging all financial support and any other relevant financial or non-financial competing interests.<br><br>This statement is required for submission and **will appear in the published article** if the submission is accepted. Please make sure it is accurate and that any funding sources listed in your Funding Information later in the submission form are also	CONFLICT OF INTEREST<br>19th September 2024<br>Title: First Time Detail Virulence and antibiogram profiling of Pseudomonas aeruginosa isolated from apparently healthy Japanese quail (Coturnix japonica) in Bangladesh<br>DECLARATION AND STATEMENT<br>We know of no conflicts of interest associated with the publication. We declare that this manuscript is original and has not been submitted to anywhere else for publication. The manuscript nicely falls under the scope of your journal.<br><br>Sincerely yours,<br><br>Mahbubul Pratik Siddique, PhD<br>Corresponding author<br>and<br>Professor<br>Department of Microbiology and Hygiene

declared in your Financial Disclosure statement.<br><br>View published research articles from *PLOS ONE* for specific examples.<br><br>**NO authors have competing interests**<br><br>Enter: *The authors have declared that no competing interests exist.*<br><br>**Authors with competing interests**<br><br>Enter competing interest details beginning with this statement:<br><br>*I have read the journal's policy and the authors of this manuscript have the following competing interests: [insert competing interests here]*<br><br>* typeset	Bangladesh Agricultural University<br>Mymensingh-2202<br>Cell Phone: +880-1751-945123<br>Email: mpsiddique@bau.edu.bd<br>https://www.researchgate.net/profile/Mahbubul-Siddique<br>https://scholar.google.com/citations?user=YmxVEzEAAAAJ&hl=en
**Ethics Statement**<br><br>Enter an ethics statement for this submission. This statement is required if the study involved:<br><br>• Human participants<br>• Human specimens or tissue<br>• Vertebrate animals or cephalopods<br>• Vertebrate embryos or tissues<br>• Field research<br><br>Write "N/A" if the submission does not require an ethics statement.<br><br>General guidance is provided below. Consult the submission guidelines for detailed instructions. **Make sure that all information entered here is included in the Methods section of the manuscript.**	Ethical Statement<br>All the animal experimental procedures were approved by the Ethical Approval Committee, Bangladesh Agricultural University, Mymensingh-2202. All the animal was handled during sample collection in compliance with the local animal welfare regulations and maintained according to standard protocols. The approval number is “AWEEC/BAU/2023(73), Date: 24.12.23.

Powered by Editorial Manager® and ProduXion Manager® from Aries Systems Corporation

**Format for specific study types**<br><br>**Human Subject Research (involving human participants and/or tissue)**<br>• Give the name of the institutional review board or ethics committee that approved the study<br>• Include the approval number and/or a statement indicating approval of this research<br>• Indicate the form of consent obtained (written/oral) or the reason that consent was not obtained (e.g. the data were analyzed anonymously)<br><br>**Animal Research (involving vertebrate animals, embryos or tissues)**<br>• Provide the name of the Institutional Animal Care and Use Committee (IACUC) or other relevant ethics board that reviewed the study protocol, and indicate whether they approved this research or granted a formal waiver of ethical approval<br>• Include an approval number if one was obtained<br>• If the study involved *non-human primates*, add *additional details* about animal welfare and steps taken to ameliorate suffering<br>• If anesthesia, euthanasia, or any kind of animal sacrifice is part of the study, include briefly which substances and/or methods were applied<br><br>**Field Research**<br><br>Include the following details if this study involves the collection of plant, animal, or other materials from a natural setting:<br>• Field permit number<br>• Name of the institution or relevant body that granted permission	
**Data Availability**<br><br>Authors are required to make all data underlying the findings described fully available, without restriction, and from the time of publication. PLOS allows rare exceptions to address legal and ethical concerns. See the PLOS Data Policy and FAQ for detailed information.	Yes - all data are fully available without restriction

A Data Availability Statement describing where the data can be found is required at submission. Your answers to this question constitute the Data Availability Statement and **will be published in the article**, if accepted.<br><br>**Important:** Stating 'data available on request from the author' is not sufficient. If your data are only available upon request, select 'No' for the first question and explain your exceptional situation in the text box.<br><br>Do the authors confirm that all data underlying the findings described in their manuscript are fully available without restriction?	
**Describe where the data may be found in full sentences. If you are copying our sample text, replace any instances of XXX with the appropriate details.**<br><br>• If the data are **held or will be held in a public repository**, include URLs, accession numbers or DOIs. If this information will only be available after acceptance, indicate this by ticking the box below. For example: *All XXX files are available from the XXX database (accession number(s) XXX, XXX.)*.<br>• If the data are all contained **within the manuscript and/or Supporting Information files**, enter the following: *All relevant data are within the manuscript and its Supporting Information files.*<br>• If neither of these applies but you are able to provide **details of access elsewhere**, with or without limitations, please do so. For example:<br><br>*Data cannot be shared publicly because of [XXX]. Data are available from the XXX Institutional Data Access / Ethics Committee (contact via XXX) for researchers who meet the criteria for access to confidential data.*<br><br>*The data underlying the results presented in the study are available from (include the name of the third party*	All relevant data are within the manuscript and its Supporting Information files

and contact information or URL).<br>• This text is appropriate if the data are owned by a third party and authors do not have permission to share the data.<br><br>* typeset	
Additional data availability information:	Tick here if your circumstances are not covered by the questions above and you need the journal’s help to make your data available.

Manuscript
Click here to access/download;Manuscript;PA_Manuscript.docx

First Time Detail Virulence and antibiogram profiling of *Pseudomonas aeruginosa* isolated from apparently healthy Japanese quail (*Coturnix japonica*) in Bangladesh

[ǂ]Alamgir Hasan, [ǂ]Md Tanjir Ahmmed, Bushra Benta Rahman Prapti, Aminur Rahman, Tasnim Islam, Chandra Shaker Chouhan, A. K. M. Anisur Rahman, and Mahbubul Pratik Siddique*

*Department of Microbiology and Hygiene, [†]Department of Medicine, Bangladesh Agricultural University, Mymensingh-2202

[1]Corresponding author: mpsiddique@bau.edu.bd

[ǂ] these authors equally contributed

Abstract

Pseudomonas aeruginosa (*P. aeruginosa*) is the predominant species associated with global poultry infections, particularly affecting Japanese quail (*Coturnix japonica*), characterized by its elevated transmissibility and mortality across all age groups. The objectives of this study were to isolate and identify *P. aeruginosa* from Japanese quails, identify risk factors, detect virulence genes and antibiotic resistance. Sampling oral and rectal swabs from 110 quail birds) across Mymensingh, Jamalpur, Gazipur and Narsingdi districts (n=110x2=220, we used cultural, morphological, biochemical, and polymerase chain reaction (PCR) methods for bacterial isolation and identification. Antibiotic sensitivity was assessed through disc diffusion against 18 commonly used antibiotics, and virulence genes were detected through PCR. We used multivariable logistic regression analysis to identify risk factors for *P. aeruginosa* infection. Both conventional and PCR methods revealed 25 (22.73%) quail birds as positive for *P. aeruginosa*. In Narsingdi, quail birds were found to be five times more likely to harbour *Pseudomonas* spp. (OR: 5.02; 95% CI:1.34–18.84) compared to those in Mymensingh Sadar. Quail birds younger than eight weeks had nearly six times higher odds (OR: 5.93; 95% CI: 1.96-17.91) of carrying *Pseudomonas* spp. compared to their older counterparts. Similarly, female quail birds showed almost four times higher odds of harbouring *Pseudomonas* spp. (OR: 3.77; 95% CI: 1.30-10.93) than male birds. All 25 *P. aeruginosa* isolates exhibited multi drug-resistance (MDR) pathogen, showing resistance to Aztreonam, Doxycycline, Chloramphenicol, and Florfenicol, yet maintaining susceptibility to Meropenem, Ceftriaxone, Cephradine, Streptomycin, Neomycin, Gentamycin, Cotrimoxazole, Ciprofloxacin, and Levofloxacin. Virulence gene distribution revealed consistent presence of *exo*A and *rhl*R in all isolates, while *exo*S, *exo*Y, *rhl*I, *rhl*AB, and *rhl*R showed variable distribution. No isolate tested positive for *exo*U, *las*I and *las*A genes. Our findings highlight the prevalence of multi-drug-resistant and virulent *P. aeruginosa* in apparently healthy quail birds, reared in

Mymensingh and Dhaka divisions of Bangladesh. This emphasizes the urgent need for a comprehensive ‘One Health’ approach to proactively address and mitigate the potential for future disasters caused by this organism in quail and human populations.

Key Words: Swab, Isolation, Risk factors, MD, Antibiogram, PCR

Introduction

Since early 1980s, poultry farming has been emerged as an industry in Bangladesh, and the majority of these poultry are indigenous chickens and ducks, which showed no satisfactory performance [1]. Apart from native poultry, people living in rural and semi-urban areas want a particular kind of bird that can be raised with minimal effort and yield greater financial returns in a short amount of time [2]. The Japanese quail (*Coturnix coturnix japonica*) has various characteristics for profitable farming, such as, higher growth rate, earlier sexual maturity, higher rate of egg production, close succession (3-4 generations per year) and less hatching period [3]. Moreover, due to small size and stout body configuration, it attributes to the less feed cost, less space requirements and most importantly, less vulnerability to common infectious diseases [3]. High potential exists for the development of Japanese quail eggs as a less expensive source of protein, particularly in developing nations [4].

The sustainability of quail farming, like that of other poultry, relies on effective management, breeding, feeding, and the implementation of disease prevention, control, and treatment measures. Japanese quail are susceptible to a variety of bacterial infections [5]. Colibacillosis, colisepticemia, fowl cholera, fowl typhoid, and salmonellosis are bacterial illnesses that have been observed in Japanese quail [6]. However, *Pseudomonas* infection in Japanese quail has been looked for unknown reason [7]. Martinez-Laorden *et al*. (2023) reported *Pseudomonas* spp. as the most prevalent bacterium in Japanese quail [8]. Again, *Pseudomonas aeruginosa* is the most prevalent species of the genus *Pseudomonas* that impact quails, leading to significant financial losses, elevated morbidity, and elevated mortality rates [9-10].

The bacterium *Pseudomonas aeruginosa*, a Gram-negative, motile, opportunistic bacterium, is prevalent in the hospital and other environments and it is a normal component of the flora of both immune compromised hosts and healthy people [11]. It causes nosocomial and ventilator-

associated infections with a high mortality [12]. *P. aeruginosa* possesses various virulence factors like Pili, exoenzyme S, LasB elastase, exotoxin A, and Phospholipase C, whereas the initiation of chronic infections involves alginate and siderophores [13]. Among the five protein secretion systems (type I, II, III, V, and the latest type VI), *P. aeruginosa* frequently employs these systems to release proteinaceous virulence factor [14-15]. Notably, the type III secretion system enables the direct injection of exotoxins into host cells upon contact, subverting host cell defences and signalling pathways [16]. Bifunctional proteins ExoT and ExoS, possessing ADP-ribosyl transferase and GTPase activating domains, impact the assembly of the actin cytoskeleton in response to extracellular signals by acting on Ras-related GTP-binding proteins like Rho, Rac, and Cdc42 [17]. Additionally, Exotoxin A inhibits protein synthesis by ADP-ribosylating eukaryotic elongation factor 2 [18]. ExoU exhibits phospholipase activity, rupturing eukaryotic membranes upon entering the cytoplasm, while ExoY possesses adenylate cyclase activity [19]. Moreover, *P. aeruginosa* utilizes quorum sensing (QS), a cell-density-dependent mechanism, to regulate the production of various virulence factors [20]. The mechanisms of disease occurrence also involve the bacterium's ability to form biofilms, which contribute to the persistence of *P. aeruginosa* in various environments, making eradication challenging [21-22] Infections caused by *P. aeruginosa* include pneumonia, urinary tract, wound and soft tissue infections.

Multidrug resistance has been increased globally, constituting a public health threat. Recent investigations have highlighted the emergence of multidrug-resistant bacterial pathogens from diverse sources, emphasizing the necessity for judicious antibiotic use. Furthermore, routine antimicrobial susceptibility testing is crucial for identifying appropriate antibiotics and screening for the emergence MDR strains [21, 23-27] preventive measures for infectious diseases are largely lacking, and the predominant approach to control such diseases relies heavily on antimicrobial therapy. However, it is crucial to recognize that AMR poses a

significant public health emergency in both human and veterinary medicine [28]. The widespread overuse of antibiotics, used for growth-promotion, metaphylactic, and prophylactic purposes in both human medicine and animal husbandry, has led to the emergence and spread of antibiotic-resistant bacteria and resistance genes. This exacerbates the risks to public health and the environment [21,29]. The impact of antimicrobial resistance include treatment failures, resulting in economic loses, and the potential to serve as a reservoir of resistant bacteria. Therefore, the selection of appropriate antimicrobial agents should be a top priority, emphasizing the need for judicious and responsible use to mitigate the adverse consequences associated with antimicrobial resistance. Identifying disease-causing agents in commercial quail farms is crucial for developing sustainable farming practices [5]. While conventional methods (cultural, morphological and biochemical) are important for bacterial detection, confirmatory diagnosis necessitates the use of polymerase chain reaction (PCR) and virulence gene detection. Existing reports on quail diseases in Bangladesh primarily focus on clinical signs and histopathology [5], and antimicrobial resistance [18], with a notable gap in molecular-based studies for agent and virulence gene detection.

Therefore, this research aims to isolate and identify *P. aeruginosa* from Japanese quails, identify risk factors, detect virulence genes and antibiotic resistance in the bacterial isolates.

Materials and methods

Ethical Statement

All the animal experimental procedures were approved by the Ethical Approval Committee, Bangladesh Agricultural University, Mymensingh-2202. All the animal was handled during sample collection in compliance with the local animal welfare regulations and maintained according to standard protocols. The approval number is "AWEEC/BAU/2023(73), Date: 24.12.23.

Sample Collection

Samples were collected from apparently healthy live birds in two commercially recognized poultry divisions belonging to Dhaka (Narsingdi and Gazipur districts) and Mymensingh (Mymensingh and Jamalpur districts) (**Fig 1**). A total of 220 samples (110 oral and 110 cloacal swabs) were collected in Luria Bertani (LB) broth from 110 apparently healthy quails from different quail shops (quail selling points) situated in Mymensingh Sadar [n= 60 (30 male + 30 female)], Jamalpur [n= 20 (10 male + 10 female)], Narsingdi [n= 20 (10 male + 10 female)], and Gazipur [n= 10 (5 male +5 female)].

Fig 1. Sampling areas, map showing Narsingdi and Gazipur districts (Dhaka division) and Mymensingh and Jamalpur districts (Mymensingh division) [map was created with ArcGIS version 10.3 (ESRI, Redlands, CA)]

Bacterial Strains, Media and Growth

The primary enrichment was made in Luria-Bertani (LB) broth (Hi-Media, India), Nutrient broth (Hi-Media, India), and Brain heart infusion (BHI) broth. Subsequently, cultured onto LB agar (Hi-Media, India), BHI agar ((Hi-Media, India), Nutrient agar (Hi-Media, India), Tryptic

soy (TSA) agar (Hi-Media, India), MacConkey (Hi-Media, India) agar and Cetrimide agar (Hi-Media, India) as selective media. Furthermore, suspected colonies were streaked on 10% Bovine Blood (BB) agar and incubation at 37°C for 24 hours. The morphological and staining properties of the isolated bacteria were documented by the Gram's staining method through microscopic observations (100X), utilising a single pure colony obtained from a *P. aeruginosa* subculture. To confirm *P. aeruginosa*, indole synthesis, methyl red test, Voges-Proskauer test, hydrogen sulphide production, oxidase and catalase tests, and sugar fermentation tests were used. The Motility Indole Urease (MIU) test was also utilized to examine the organism's motility.

Genomic DNA Extraction and PCR Amplification

Genomic DNA extraction from the isolates was performed through the boiling and thawing technique [30]. Subsequently, NanodropTM (Thermo Fisher Scientific, USA) was used for both quantifying and assessing the quality of the extracted DNA. To standardise the 16S rDNA oligonucleotides, bacterial DNA was mixed yielding an average concentration of 130 ng/µl and a purity of 2.10. The PCR reaction volume of 20 µl comprised 3 µl DNA template, 1 µl each of forward and reverse primers, 10 µl of PCR Master Mix, 2X (Promega, USA), and 5.5 µl of nuclease free water. Table 1 provides details on the primers and techniques used for the PCR reactions [31]. Analysis of the PCR end products was conducted using a 1.5% agarose gel. Post-electrophoresis, the gel was immersed in an ethidium bromide solution (concentration approximately 0.2-0.5 µg/mL) for 10 minutes and subsequently visualised using a UV transilluminator.

Virulence Profile of P. aeruginosa

The primer sets for PCR-based detection of the virulence-associated genes, along with the amplification protocols, were chosen from previously published papers with slight

modifications, as summarized in **Table 1.** The visualization of PCR end products followed the procedure mentioned earlier.

Table 1 List of primers, with oligonucleotide sequences, amplification conditions, amplicon size and references, which were used to detect *Pseudomonas* at genus and species level, as well as to detect the virulence genes by PCR

**Gene**	**Sequence (5'-3')**	**Thermal profile**										**Amplicon size (bp)**	**References**
		Initial denaturation		Denaturation		Annealing		Elongation		Final extension			
		Tem	Time	Tem	Time	Tem	Time	Tem	Time	Tem	Time		
16S rDNA GS	F: GACGGGTGAGTAATGCCTA	95°C	2 m	94°C	20 s	54°C	20 s	72°C	40 s	72°C	10 m	618	[31]
	R: CACTGGTGTTCCTTCCTATA												
16S rDNA SS	F: GGGGGATCTTCGGACCTCA	95°C	2 m	94°C	20 s	58°C	20 s	72°C	40 s	72°C	10 m	956	
	R: TCCTTAGAGTGCCCACCCG												
*exo*A	F: GACAACGCCCTCAGCATCACCAGC	94°C	2 m	94°C	1 m	58°C	1 m	72°C	30 s	72°C	5 m	396	[32]
	R: CGCTGGCCCATTCGCTCCAGCGCT												
*exo*S	F: GCGAGGTCAGCAGAGTATCG	94°C	2 m	94°C	30 s	58°C	30 s	72°C	20 s	72°C	5 m	118	
	R: TTCGGCGTCACTGTGGAT												
*exo*T	F: AATCGCCGTCCAACTGCATGCG	94°C	2 m	94°C	20 s	58°C	1 m	72°C	20 s	72°C	5 m	152	
	R: TGTTCGCCGAGGTACTGCTC												
*exo*Y	F: CGGATTCTATGGCAGGGAGG	94°C	2 m	94°C	1 m	58°C	1 m	72°C	30 s	72°C	5 m	289	
	R: GCCCTTGATGCACTCGACCA												
*exo*U	F: ATGCATATCCAATCGTTG	94°C	2 m	94°C	1 m	54°C	1 m	72°C	2 m	72°C	10 m	2000	
	R: TCATGTGAACTCCTTATT												
*rhl*AB	F: TCATGGAATTGTCACAACCGC	94°C	2 m	94°C	20 s	58°C	20 s	72°C	15 s	72°C	7 m	151	
	R: ATACGGCAAAATCATGGCAAC												
*rhl*R	F: CAATGAGGAATGACGGAGGC	94°C	2 m	94°C	1 m	50°C	30 s	72°C	45 s	72°C	7 m	730	
	R: GCTTCAGATGAGGCCCAGC												
*rhl*I	F: CTTGGTCATGATCGAATTGCTC	94°C	2 m	94°C	1 m	50°C	30 s	72°C	40 s	72°C	7 m	625	
	R: ACGGCTGACGACCTCACAC												
*las*I	F: ATGATCGTACAAATTGGTCGGC	94°C	2 m	94°C	1 m	50°C	30 s	72°C	35 s	72°C	7 m	605	
	R: GTCATGAAACCGCCAGTCG												
*las*A	F: CGCCATCCAACCTGATGCAAT	94°C	2 m	94°C	1 m	60°C	1 m	68°C	30 s	72°C	7 m	514	
	R: AGGCCGGGGTTGTACAACGGA												

Legend: bp: base pair; Tem = Temperature; m = minute; s = second; GS = genus-specific; SS = Species-specific

Antibiotic Susceptibility of P. aeruginosa

The antibiogram test utilized commonly used antibiotics at the field level through the disc diffusion method [33]. In brief, the turbidity of all isolates was adjusted to a 0.5% McFarland standard by culturing them in LB broth (incubated at 37℃ for approximately 4 hours in a shaking incubator). Subsequently, the isolates were inoculated onto MHA (Liofilchem, Italy). Following inoculation, the antibiotic discs were placed and the entire setup was incubated at 37℃ for 18 hours to measure the diameter of the inhibition zone. To determine sensitivity or resistance of the isolates the Clinical and Laboratory Standards Institute (CLSI, 2019) standard interpretation chart for the zone of inhibition was referenced [34]. The antibiotic discs used in this study included Beta lactam Ampicillin G (AMP, 25 µg/disc), Ceftriaxone (CTR, 30 µg/disc), Cefuroxime (CXM, 30 µg/disc), Cephradine (CE, 30 µg/disc), Meropenem (MEM, 10 µg/disc), Aztreonam (AT, 30 µg/disc) Non-beta lactam Streptomycin (S, 10 µg/disc), Gentamycin (GEN, 30 µg/disc), Neomycin (N, 30 µg/disc), Doxycycline (DO, 30 µg/disc), Oxytetracycline (O, 30 µg/disc), Cotrimoxazole (COT, 25 µg/disc), Cefoxitin (FOX, 30 µg/disc), Chloramphenicol (C, 30 µg/disc), Florfenicol (FFC, 25 µg/disc), Nalidixic acid (NA, 30 µg/disc), Ciprofloxacin (CIP, 5 µg/disc), and Levofloxacin (LEV 5 µg/disc). Among the discs, CTR, AT, GEN, and O were purchased from HiMedia Laboratories Private Limited (Maharashtra, India) and rest were from Oxoid Limited (Hampshire, UK).

Statistical Analysis

The data on location, age, gender, sample site, and test results, were first entered into Excel 2010 and then transferred to R 4.3.2 [35] for analysis. A quail bird was considered as affected if it tested positive for *Pseudomonas* spp. either in rectal or oral swabs. Continuous variable age was converted into categorical variable (≤ 8 and > 8 weeks). The prevalence of *Pseudomonas* spp. across different variables was estimated using the ‘tabpct’ function of the

R ‘epiDisplay’ package [36]. The association between *Pseudomonas* spp. infection and all explanatory variables was first investigated using univariable logistic regression model. Following that, multivariable logistic regression analysis was performed, incorporating only those explanatory variables with a univariable p-value of ≤ 0.2. Prior to conducting the multivariable logistic regression analysis, the variation inflation factor (VIF) was used to assess multicollinearity among the explanatory variables, with a cut-off level set at < 5. A backward model selection strategy was implemented for the final model selection, and the overall fit of the model was evaluated using the Hosmer-Lemeshow test.

The correlation coefficient between phenotypic and genotypic resistance was calculated using the 'cor' function in R 4.3.2 [35].

Sequencing and Phylogenetic Analysis

For the sequencing of the 16S rRNA gene, two isolates of *Pseudomonas* spp. (PCR detection up to genus level; 618 bp) were selected, ensuring coverage from both divisions (Dhaka and Mymensingh). Additionally, two isolates of *P. aeruginosa* (PCR detection at species level; 956 bp) were chosen from each division. The PCR was conducted using the aforementioned primer sets, reaction mixtures and thermal profiles. Successful amplifications were confirmed through 1.5% agarose gel electrophoresis and staining with ethidium bromide (EtBr) (Research-Lab Fine Chem Industries, Mumbai, 400 002, India), followed by visualization under a UV trans-illuminator (Biometra, Germany).

The amplified PCR products were purified using the QIAamp PCR product purification kit (QIAgen GmbH, Hilden, Germany). Subsequently, the purified PCR products underwent commercial sequencing at the National Institute of Biotechnology, Savar, Dhaka, Bangladesh, using the Sanger Dideoxy sequencing method. For sequencing a single amplicon, both forward and reverse primers were used. Raw sequence data were aligned, considering the forward

sequence and the reverse complement of reverse sequence, and then edited using CLC sequence viewer v8 (**http://www.clcbio.com**) and BioEdit v7.2 [37] sequence editing and analysis tools. To determine sequence similarities with sequences already deposited in the NCBI GenBank, the edited sequences were subjected to BLAST. The GenBank accession numbers assigned to the present isolates are OR617385 to OR617388.

To assess the evolutionary relationships, a total of 56 partial and complete 16S rRNA gene sequences of *P. aeruginosa* were retrieved from NCBI GenBank. These sequences represent data from every division and source in Bangladesh (at least one from each division and source), neighbouring countries (India, Pakistan, China, Japan, Thailand) and at least one sequence from each continent. The 56 downloaded sequences, along with four of our sequences, were aligned using MUSCLE within MEGA11: Molecular Evolutionary Genetics Analysis version 11 software [38] package and exported in MEGA format. Finally, the MEGA11 software package was used to construct a phylogenetic tree employing 1000 Bootstrap replications. The tree was further annotated using the Interactive Tree Of Life (iTOL) v5: an online tool for phylogenetic tree display and annotation [39].

Results

Pseudomonas aeruginosa, a significant bacterial pathogen, poses potential threats to both animal and human health. In this study, our primary objectives were to investigate the prevalence, antibiotic resistance patterns, and virulence gene profiles of *P. aeruginosa* in apparently healthy quail birds in Bangladesh. We aimed to explore potential risk factors associated with the presence of this pathogen.

Phenotypic characteristics of the recovered isolates

The bacterial isolates showed robust growth in LB broth, characterized by dense turbidity and the formation of a pellicle. In nutrient broth at 37°C for 24 hours under aerophilic conditions, the culture displayed turbidity with a green pigment, while BHI broth resulted in turbidity only (data not shown). On LB agar, the organism demonstrated an earthy odour and irregular swarming growth. Conversely, on BHI agar, the isolates produced slimy mucoid colonies. Nutrient agar revealed small, irregular bluish-green colonies with a sweet odour. Cetrimide agar exhibited circular, smooth, small to medium-sized, mucoid greenish-blue opaque colonies. MacConkey agar displayed colorless, circular smooth, mucoid transparent colonies, while blood agar showed, irregularly edged circular flat smooth greyish non-haemolytic white colonies. Gram staining identified the organism as Gram-negative, rod-shaped, and arranged singly, in pairs, or in short chains. Furthermore, the Motility Indole Urease/Urea (MIU) medium test confirmed the organism's motility, but it tested negative for indole and urease (data not shown). Suspected *Pseudomonas* spp. was found to be positive for Dextrose, Maltose, and Sucrose fermentation with acid and gas production, while testing negative for Lactose and Mannitol. Additionally, the organism exhibited a positive Methyl Red (MR) test but tested negative for the Indole and Voges-Proskauer tests (data not shown).

Confirmation by PCR

Genus level identification was confirmed by the presence of a positive band at 618 base pairs during gel electrophoresis (**Fig 2a**). Subsequently, specific detection of *P. aeruginosa* was confirmed by the presence of a 956 bp rRNA gene product (**Fig 2b**).

Fig 2. Agarose gel electrophoresis results of PCR amplification targeting 16S rRNA gene for the genus- and species-specific detection of *Pseudomonas* spp. and *P. aeruginosa*, respectively. (a) genus-specific PCR amplification of *Pseudomonas* spp. and positive bands appeared at 618 bp; (b) species-specific detection of *P. aeruginosa*, with amplicon size of 956 bp. In both cases, Lane M: 100 bp DNA ladder; NC: negative control; Lane 1-9/10: positive isolates.

Prevalence of P. aeruginosa

Of the 110 quail birds' rectal and oral swabs, cultural, Gram staining, and biochemical tests showed the presence of *Pseudomonas* spp. In 25 (22.73%) samples (Table 2). Specifically, *Pseudomonas* spp. were detected in 19.09% of rectal swabs, 6.36% of oral swabs, and 2.73% of samples obtained from both rectal and oral swabs (**Table 2**). Moreover, all isolates conventionally characterized were also confirmed positive in PCR amplification using specific primer sets.

Table 2 Prevalence of the isolated bacteria (culture and PCR-based)

District	Species	Sample type	Total samples	Total positive sample	Prevalence %	Overall prevalence
Mymensingh Sadar (n= 60)	Male	Oral swab	30	0 (60)	0	Male = 9 (55), 16.36% Female = 16 (55), 29.09 %
		Rectal Swab	30	2 (60)	6.67	
	Female	Oral swab	30	3 (60)	5	
		Rectal Swab	30	6 (60)	15	
Jamalpur Sadar (n= 20)	Male	Oral swab	10	0 (20)	0	
		Rectal Swab	10	2 (20)	10	
	Female	Oral swab	10	1 (20)	10	
		Rectal Swab	10	2 (20)	15	
Narsingdi Sadar (n= 20)	Male	Oral swab	10	1 (20)	5	
		Rectal Swab	10	3 (20)	20	
	Female	Oral swab	10	1 (20)	5	
		Rectal Swab	10	2 (20)	15	
Gazipur Sadar (n= 10)	Male	Oral swab	05	0 (10)	0	
		Rectal Swab	05	1 (10)	10	
	Female	Oral swab	05	0 (10)	0	
		Rectal Swab	05	1 (10)	10	
110 birds			**220**	**25 (110x2=220)**		**22.73% (25/110 birds)**

Risk Factors Analysis for Isolated P. aeruginosa

The multivariable analysis incorporated four variables that showed associations with *P. aeruginosa* infection in quail. at p-values ≤0.2 in the univariable analysis (**Table 3**). The final multivariable logistic regression model identified three specific variables as risk factors (**Table 4**) for *P. aeruginosa* infection in quail. In Narsingdi, quail birds were found to be five times more likely to harbour *Pseudomonas* spp. (OR: 5.02; 95% CI:1.34–18.84) compared to those in Mymensingh Sadar. Quail birds younger than eight weeks had nearly six times higher odds (OR: 5.93; 95% CI: 1.96-17.91) of carrying *Pseudomonas* spp. compared to their older counterparts. Similarly, female quail birds showed almost four times higher odds of harbouring *Pseudomonas* spp. (OR: 3.77; 95% CI: 1.30-10.93) than male birds.

Table 3 Univariable association between *Pseudomonas* spp. infection and explanatory variables

Risk factors	Category level	*Pseudomonas*		Prevalence (%)	OR	95% CI	P-value
		+ve	-ve				
District*	Mymensingh Sadar	10	50	16.7	Ref	-	-
	Jamalpur Sadar	5	15	25.0	1.67	0.49-5.64	0.411
	Gazipur Sadar	2	8	20.0	1.25	0.23-6.79	0.800
	Narsingdi Sadar	8	12	40.0	3.33	1.08-10.25	0.036
Sex*	Male	7	46	13.2	Ref	-	-
	Female	18	39	31.6	3.03	1.15-8.01	0.025
Age* (weeks)	> 8	9	57	13.6	Ref	-	-
	≤ 8	16	28	36.4	3.62	1.42-9.21	0.007
Sample site*	Market	10	49	16.9	Ref	-	-
	Farm	15	36	29.4	2.04	0.82-5.06	0.124

+ve: Positive; -ve: Negative; OR: Odds ratio; CI: 95% confidence interval; * Candidate variable for multivariable analysis

Table 4 Risk factors identified in the final multivariable model for *Pseudomonas* spp. infection in quail birds

Risk factors	Category level	OR	95% CI	P-value
District*	Mymensingh Sadar	Ref	-	-
	Jamalpur Sadar	2.26	0.59-8.68	0.236
	Gazipur Sadar	3.01	0.47-619.50	0.247
	Narsingdi Sadar	5.02	1.34-18.84	0.017
Sex*	Male	Ref	-	-
	Female	3.77	1.30-10.93	0.014
Age* (week)	> 8	Ref	-	-
	< 8	5.93	1.96-17.91	0.002

OR: Odds ratio; CI: 95% confidence interval; * Candidate variable for multivariable analysis

Distribution of Virulence Genes of P. aeruginosa

The presence of virulence genes among the 25 isolates was as follows: *exo*A (100%; amplicon size 396 bp), *exo*S (88%; amplicon size 118 bp), *exo*T (64%; amplicon size 152 bp), *exo*Y (84%; amplicon size 289 bp), *rhl*AB (76%; amplicon size 151 bp), *rhl*R (100%; amplicon size 730 bp), *rhl*I (64%; amplicon size 625 bp). Notably, none of the isolates tested positive for

exoU, *las*I and *las*A. The individual profile of virulence genes for each isolate is represented in **Table 5**, and the overall prevalence is visualized in **Fig 3**. Further, the PCR amplification results for each positive virulence gene is shown **Fig 4.**

Table 5 Isolate-wise virulence genes distribution of *P. aeruginosa*

**Location**	**Isolate's code**	**Virulence associated genes**						
		***exo*A**	***exo*S**	***exo*T**	***exo*Y**	***rhl*AB**	***rhl*R**	***rhl*I**
Gazipur	PD103	1	1	1	1	1	1	1
Gazipur	PD107	1	1	1	1	0	1	1
Jamalpur	PM61	1	1	1	1	1	1	0
Jamalpur	PM63	1	1	1	1	1	1	1
Jamalpur	PM65	1	1	1	1	1	1	0
Jamalpur	PM71	1	1	1	1	1	1	1
Jamalpur	PM72	1	1	1	1	1	1	1
Mymensingh	PM1	1	1	1	1	1	1	1
Mymensingh	PM3	1	1	1	1	0	1	1
Mymensingh	PM6	1	1	1	0	1	1	1
Mymensingh	PM7	1	1	1	1	1	1	1
Mymensingh	PM8	1	1	1	1	1	1	1
Mymensingh	PM15	1	1	1	1	0	1	0
Mymensingh	PM19	1	1	1	1	1	1	1
Mymensingh	PM22	1	1	1	1	1	1	0
Mymensingh	PM44	1	1	1	0	1	1	0
Mymensingh	PM56	1	0	1	1	1	1	1
Mymensingh	PM57	1	1	1	1	0	1	0
Narsingdi	PD81	1	1	1	1	1	1	1
Narsingdi	PD82	1	1	1	1	1	1	1
Narsingdi	PD84	1	0	1	0	1	1	1
Narsingdi	PD86	1	1	1	1	0	1	0
Narsingdi	PD88	1	1	1	1	1	1	0
Narsingdi	PD91	1	1	1	1	1	1	1
Narsingdi	PD100	1	0	0	0	0	1	0

Note: *exo*U, *las*I and *las*A were not found, so excluded from this table.

Fig 3. Frequency distribution of virulence genes in isolated *P. aeruginosa*

Fig 4. Virulence genes detection in PCR confirmed *P. aeruginosa* isolates. [A] *exo*A (396 bp), [B] *exo*S (118 bp), [C] *exo*T (152 bp), [D] *exo*Y (289 bp), [E] *rhl*AB (151 bp), [F] *rhl*R (730 bp), and [G] *rhl*I (625 bp). However, there were no *exo*U, *las*I and *las*A found in any isolates. In all cases, the Lane M= 100 bp DNA ladder; NC= negative control; and clear and consistent band showing lanes indicated positive isolates

Antimicrobial susceptibility testing of P. aeruginosa

The antibiotic resistance profile of *P. aeruginosa* was illustrated in the **Fig 5**. The results revealed that 92% of the isolates showed resistance to ampicillin, all isolates showed 100% resistance to 1st generation cephalosporin (Cephradine). Furthermore, resistance to other β-lactams varied, with cefoxitin displaying 76% resistance, while ceftriaxone and meropenem exhibited 100% susceptibility. Within the aminoglycoside group, streptomycin showed 28% susceptibility and 72% intermediate resistance. Conversely, neomycin, gentamycin, cotrimoxazole, chloramphenicol, florfenicol, ciprofloxacin, levofloxacin demonstrated 100% susceptibility. On the other hand, the tetracycline group and quinolone group including oxytetracycline, doxycycline, and nalidixic acid, showed 92% and 100% resistance respectively. The antibiotic resistance patterns for each isolate were showed in **Fig 5.**

Fig 5. Isolate-wise antibiotic resistance patterns of the isolated *P. aeruginosa* from oral and rectal swabs of quail birds

Moreover, **Fig 6** illustrated the isolate-wise distribution of virulence genes and the count of phenotypic antimicrobial resistance in *P. aeruginosa* isolates from quail birds in both the Mymensingh and Dhaka divisions. Notably, we observed a weak positive association (r=0.11) between phenotypic and genotypic resistance **(Fig 6)**

Fig 6. Isolate-wise with total number-based virulence genes occurrence and phenotypic antimicrobial resistance in the *P. aeruginosa* isolates

Phenotypic Multi Drug-Resistance (MDR) Nature of P. aeruginosa Isolates

All *P. aeruginosa* isolates obtained from rectal and oral swabs of quail birds displayed a multidrug-resistant (MDR) phenotype. The 25 isolates from both rectal and oral swabs

exhibited resistance to Aztreonam, Doxycycline, Chloramphenicol, Florfenicol, and Cefuroxime (ATM, DO, C, FFC, CXM), out of the 18 tested antibiotics. Among the 21 rectal swab isolates, nine exhibited resistances to a broader spectrum of nine antibiotics including Aztreonam, Doxycycline, Chloramphenicol, Florfenicol, Cefuroxime, Oxytetracycline, Ampicillin, Nalidixic acid, and Cefoxitin (ATM, DO, C, FFC, CXM, OT, AMP, NA, FOX,) (**Table 6**). In contrast, all 25 isolates were found to be susceptible to nine antibiotics, namely Meropenem, Ceftriaxone, Cephradine, Streptomycin, Neomycin, Gentamycin, Cotrimoxazole, Ciprofloxacin, and Levofloxacin.

Table 6 Frequency distribution of phenotypic resistance and virulence gene profiles of *P. aeruginosa* from rectal and oral swabs of quail birds

**Antibiotic profiles and resistance genes of isolated *P. aeruginosa* isolates**		
**Rectal swabs of quail birds**		
**Isolate**	**Resistant Phenotypes**	**Virulence genotype**
R1	ATM, DO, C, FFC, CXM, OT, AMP, NA, FOX	*exo*A, *exo*S, *exo*T, *exo*Y, *rhl*AB, *rhl*I, *rhl*R
R6	ATM, DO, C, FFC, CXM, OT, AMP, NA	*exo*A, *exo*S, *rhl*AB, *rhl*I, *rhl*R
R7#	ATM, DO, C, FFC, CXM, OT, AMP, FOX	*exo*A, *exo*S, *exo*Y, *rhl*AB, *rhl*I, *rhl*R
R8	ATM, DO, C, FFC, CXM, OT, AMP, NA, FOX	*exo*A, *exo*S, *exo*T, *exo*Y, *rhl*AB, *rhl*I, *rhl*R
R15#	ATM, DO, C, FFC, CXM, OT, AMP, FOX	*exo*A, *exo*S, *exo*T, *exo*Y, *rhl*R
R22	ATM, DO, C, FFC, CXM, OT, AMP, NA	*exo*A, *exo*S, *exo*Y, *rhl*AB, *rhl*R
R44	ATM, DO, C, FFC, CXM, OT, AMP, NA, FOX	exoA, *exo*S, *rhl*AB, *rhl*R
R56	ATM, DO, C, FFC, CXM, OT, AMP, NA, FOX	*exo*A, *exo*S, *exo*Y, *rhl*AB, *rhl*I, *rhl*R
R57	ATM, DO, C, FFC, CXM, OT, FOX	*exo*A, *exo*S, *exo*T, *exo*Y, *rhl*R
R61	ATM, DO, C, FFC, CXM, AMP, NA, FOX	*exo*A, *exo*S, *exo*Y, *rhl*AB, *rhl*R
R63	ATM, DO, C, FFC, CXM, OT, AMP, NA	*exo*A, *exo*S, *exo*T, *exo*Y, *rhl*AB, *rhl*I, *rhl*R
R65	ATM, DO, C, FFC, CXM, OT, AMP, FOX	*exo*A, *exo*S, *exo*T, *exo*Y, *rhl*AB, *rhl*R
R71	ATM, DO, C, FFC, CXM, OT, AMP, NA	*exo*A, *exo*S, *exo*T, *exo*Y, *rhl*AB, *rhl*I, *rhl*R
R82	ATM, DO, C, FFC, CXM, OT, AMP, NA, FOX	*exo*A, *exo*S, *exo*T, *exo*Y, *rhl*AB, *rhl*I, *rhl*R
R84	ATM, DO, C, FFC, CXM, OT, AMP, NA, FOX	*exo*A, *exo*T, *rhl*AB, *rhl*I, *rhl*R
R86#	ATM, DO, C, FFC, CXM, OT, AMP, NA	*exo*A, *exo*S, *exo*T, *exo*Y, *rhl*R

R88	ATM, DO, C, FFC, CXM, AMP, FOX	*exo*A, *exo*S, *exo*T, *exo*Y, *rhl*AB, *rhl*R
R91	ATM, DO, C, FFC, CXM, OT, AMP, NA	*exo*A, *exo*S, *exo*Y, *rhl*AB, *rhl*I, *rhl*R
R100	ATM, DO, C, FFC, CXM, OT, AMP, NA, FOX	*exo*A, *rhl*R
R103	ATM, DO, C, FFC, CXM, OT, AMP, NA, FOX	*exo*A, *exo*S, *exo*T, *exo*Y, *rhl*AB, *rhl*I, *rhl*R
R107	ATM, DO, C, FFC, CXM, OT, AMP, NA, FOX	*exo*A, *exo*S, *exo*Y, *rhl*I, *rhl*R
**Oral swabs of quail birds**		
**Isolate**	**Resistant Phenotypes**	**Virulence genotype**
O3	ATM, DO, C, FFC, CXM, OT, AMP, NA, FOX	*exo*A, *exo*S, *exo*T, *exo*Y, *rhl*AB, *rhl*I, *rhl*R
O7[#]	ATM, DO, C, FFC, CXM, OT, AMP, FOX	*exo*A, *exo*S, *exo*Y, *rhl*AB, *rhl*I, *rhl*R
O15[#]	ATM, DO, C, FFC, CXM, OT, AMP, FOX	*exo*A, *exo*S, *exo*T, *exo*Y, *rhl*R
O19	ATM, DO, C, FFC, CXM, OT, NA, FOX	*exo*A, *exo*S, *exo*T, *exo*Y, *rhl*AB, *rhl*I, *rhl*R
O72	ATM, DO, C, FFC, CXM, OT, AMP, NA, FOX	*exo*A, *exo*S, *exo*T, *exo*Y, *rhl*AB, *rhl*I, *rhl*R
O81	ATM, DO, C, FFC, CXM, OT, AMP, NA, FOX	*exo*A, *exo*S, *exo*Y, *rhl*AB, *rhl*I, *rhl*R
O86[#]	ATM, DO, C, FFC, CXM, OT, AMP, NA	*exo*A, *exo*S, *exo*T, *exo*Y, *rhl*R

Note: *O = Oral swabs, R= Rectal swabs, ATM = Aztreonam, DO= Doxycycline, C= Chloramphenicol, FFC= Florfenicol, OT= Oxytetracycline, AMP= Ampicillin, NA=Nalidixic acid, FOX= Cefoxitin, # Samples positive for both samples

The multiple antibiotic resistance (MAR) index analysis showed that only one isolate (PM1) had MAR index of 0.167, however, 10 isolates (PM2, 7, 10-13, 15, 17-19) had MAR index of 0.278 and 14 isolates (PM3-6, 8, 9, 14, 16, 20-25) had the MAR index of 0.333. The total isolates (n=25) were also analysed based on antimicrobial classes to represent their nature as XDR, PDR, and MARI (Table 7; Fig 7)

Table 7. *Pseudomonas aeruginosa:* antimicrobial categories and agents used to define MDR**,** XDR, PDR and MARI

Antimicrobial category	Antimicrobial agent	Number of isolates resistant	Multiple Antibiotic Resistant Index (%)
Beta lactam	Ampicillin	0	0
Cephalosporin	Ceftriaxone	0	0

	Cephradine		
	Cefoxitin	19	76
	Cefuroxime	25	100
Antipseudomonal carbapenems	Meropenem	0	0
Monobactams	Aztreonam	25	100
Aminoglycosides	Streptomycin		
	Gentamicin	0	0
	Neomycin	0	0
Tetracycline	Doxycycline	25	100
	Oxytetracycline	23	92
Sulfonamides	Cotrimoxazole		
Phenicols	Chloramphenicol	0	0
	Florfenicol	0	0
Fluoroquinolones	Ciprofloxacin	0	0
	Levofloxacin	0	0
Quinolones	Nalidixic acid	20	80

Criteria for defining MDR, XDR and PDR in *Pseudomonas aeruginosa*
MDR: non-susceptible to ≥1 agent in ≥3 antimicrobial categories.
XDR: non-susceptible to ≥1 agent in all but ≤2 categories.
PDR: non-susceptible to all antimicrobial agents listed.
http://www.ecdc.europa.eu/en/activities/diseaseprogrammes/ARHAI/Pages/public_consultation_clinical_microbiology_infection_article.aspx

Fig 7. Correlation between phenotypic and genotypic resistance

Sequencing and Phylogenetic Analysis

The phylogenetic analysis of the sequenced PCR amplification products revealed that, at the genus level, the *Pseudomonas* sp. isolates from quails in both Narsingdi (GenBank Accession No. OR617385) and Mymensingh (GenBank Accession No. OR617386) districts exhibited no evolutionary differences. Moreover, they displayed a close relationship with *Pseudomonas* isolates from milk (GenBank Accession No. MN464147; raw milk from Sylhet) and rhizosphere soil (GenBank Accession No. MG786551; source region not mentioned),

respectively. Similarly, the analysed sequenced products of *P. aeruginosa* from quails in both Narsingdi (GenBank Accession No. OR617387) and Mymensingh (GenBank Accession No. OR617388) regions clustered very closely with the *P. aeruginosa* isolates from municipal waste in the Sylhet region (GenBank Accession No. MW040069) and hospital wastes from Khulna region (GenBank Accession No. MG735679), respectively (Fig 8).

Fig 8. Phylogenetic relationship of the isolated *P. aeruginosa* from quail birds of Narsingdi and Mymensingh regions of Bangladesh. The sequenced and analysed isolates were marked by various colour.

Discussion

Our findings highlight the prevalence of multi- drug-resistant and virulent potential *P. aeruginosa* in apparently healthy quail birds reared in Mymensingh and Dhaka divisions of Bangladesh. Young female birds should be regularly screened for future surveillance. We suggested a One Health approach to proactively address and mitigate any future disasters caused by this organism in quail and human populations.

The susceptibility of young birds to *P. aeruginosa* infections is well-documented, with the potential for mechanical transmission through skin injuries or contaminated needles used in vaccinations. Factors such as the bird's immunological status and concurrent illnesses can exacerbate vulnerability [40]. This study identified PCR positive isolates of *P. aeruginosa*, in quails at a proportion of 22.73% Notably, there is a scarcity of studies on *P. aeruginosa* in poultry in Bangladesh. A recent study by Bakheet and Torra (2020) reported a 24% positivity rate in poultry [41], while [42] found a 28.3% prevalence in broiler chickens from Ismailia Governorate, Egypt. Higher prevalences were documented by Abd El-Tawab et al. (2019), who reported a 34% prevalence in broiler chickens [43]. Shahat et al. (2019) documented a 20% prevalence for *P. aeruginosa* in broiler chickens based on 16S Rdna [44]. The prevalence of *P.*

aeruginosa in quail birds in Narsingdi district was significantly higher Mymensingh. This variation may be attributed to diverse management practices, agro-climatic conditions such as rainfall, humidity, temperature, and the inherent prevalence of *P. aeruginosa* in the Narsingdi region. The significance of regional residence as a risk factor aligns with findings in human infections with *P. aeruginosa*, where the regional factor contributes to higher prevalence [45].

In terms of age, the study found that the prevalence of *P. aeruginosa* in young birds (<8 weeks old) was six times higher than in older birds (>8 weeks old). This observation is consistent with existing literature [25, 46-47]. These studies highlighted the heightened susceptibility of young chicks to *P. aeruginosa* infections. Additionally, mortality was noted to be higher in young birds [48], further emphasizing the vulnerability of this age group.

Furthermore, the study identified a higher prevalence of *P. aeruginosa* in female birds compared to male birds This finding aligns with studies on human infections, where female patients are more susceptible than males [49-50] The female reproductive hormones may contribute to increased vulnerability to *P. aeruginosa* infection [51].

Understanding the pathophysiology of opportunistic pathogens such as *P. aeruginosa* is important and characterizing the virulence factors that constitute its defensive mechanisms is instrumental in exploring novel antimicrobial strategies, especially for multidrug-resistant (MDR) strains. *P. aeruginosa* secretes various extracellular components to support its survival and enhance virulence [52]. The four effector proteins, *ExoS*, *ExoT*, *ExoU*, and *ExoY*, secreted by *P. aeruginosa* through the type III secretion system, play a pivotal role in its pathogenicity [53]. These proteins impact critical host cell activities, including signal transduction and cytoskeletal organization [54]. Wareham and Curti (2007) described ExoS and ExoT as bifunctional toxins with the ability to activate GTPase and ADP-ribosyltransferase [55]. In this study, we identified the virulence genes exoS (88%) and exoT (64%), consistent with the findings of Wareham and Curti, who noted that exoT exhibits lower ADP-ribosyltransferase

activity than exoS [55]. Moreover, *Exo*U demonstrates phospholipase activity, leading to the rupture of eukaryotic membranes upon entering the cytoplasm, while *Exo*Y possesses adenylate cyclase activity. The presence of *exo*Y (84%) in our study suggests that *P. aeruginosa* exhibited adenylate cyclase activity, while no *exo*U was detected. Additionally, the protein synthesis gene *exo*A was found to be present at 100%. Ghazaei (2021) reported a similar presence of the *exo*A virulence gene at 83.33% in animal samples [56]. The intricate quorum sensing (QS) systems of *P. aeruginosa*, particularly the conventional LuxI/R-type QS systems, LasI/R and RhlI/R, play a pivotal role in managing the synthesis of virulence factors and the formation of biofilms. According to Bakheet and Torra (2020), these systems generate and detect 3OC12-homoserine lactone and C4-homoserine lactone, respectively [41]. Interestingly, the auto inducer *las*I gene responsible for secreting key virulence tools like pyocyanin and elastase was not detected in any of the isolates in this study [57]. This contrasts with the findings of Bakheet and Torra (2020), who reported the presence of *las*I in 100% of *P. aeruginosa* strains [41]. Similarly, lasR was present in 75% of strains in studies by Kebede (2010) and Habeeb et al. (2012) [58-59]. Despite the absence of the *las*I gene in the isolates of this investigation, the presence of QS activity was confirmed through the detection of *rhl*R (100%), *rhl*I (64%), and other markers. Furthermore, *P. aeruginosa* possesses the capacity to develop antibiotic resistance through mutations in genes encoded in its DNA or via the transfer of genes across generations [58]. The presence of plasmids, integrons, and transposons carrying antibiotic-resistant genes in *P. aeruginosa* is widely recognised [46]. The multidrug-resistant features carried by *P. aeruginosa*, with the potential for transmission to other human and animal diseases, make it a particularly concerning pathogen for consumer health [48].

Notably, a substantial 92% of isolates displayed resistance to ampicillin, while all isolates exhibited 100% resistance to first-generation cephalosporin (cephradine). This aligns with the findings of Shahat *et al*. (2019) [44] and is consistent with reports by Walker et al. (2002),

Ahmed (2016), and Tartor and El-Naenaeey (2016), who highlighted high levels of resistance to tetracycline and ampicillin [60-62].

Interestingly, within the β-lactam category, cefoxitin demonstrated 76% resistance, whereas ceftriaxone and meropenem displayed complete susceptibility (100%). These results diverge from the general resistance trend observed in the β-lactam group, emphasizing the nuanced response of *P. aeruginosa* to different antibiotics within the same class. In the aminoglycoside group, streptomycin exhibited 28% susceptibility and 72% intermediate resistance. However, neomycin, gentamycin, cotrimoxazole, chloramphenicol, florfenicol, ciprofloxacin, and levofloxacin demonstrated 100% susceptibility. These findings align with Shahat et al. (2019) and support the effectiveness of ciprofloxacin and gentamycin against *P. aeruginosa* [44], as noted by Mohammad (2013) [63]. Conversely, the tetracycline and quinolone groups, including oxytetracycline, doxycycline, and nalidixic acid, exhibited 92% and 100% resistance, respectively. The high resistance to nalidixic acid (80%) aligns with the observations of [64]. Beside these alarming situations, it was also observed that most of the 96% isolates showed MARI values of more than 0.2 which indicated high risk source of antibiotic resistant bacterial contamination [65]. The findings were in close agreement with the report of Afunwa et al. (2020) [66].

Conclusion

Our findings highlight a concerning prevalence of *P. aeruginosa* in apparently healthy quail birds. This prevalence varied significantly according to sampling site, birds age and sex. Furthermore, the observed resistance of the 25 isolates to Aztreonam, Doxycycline, Chloramphenicol, Florfenicol, and Cefuroxime (ATM, DO, C, FFC, CXM) raises alarms regarding the development of multi-drug resistance in these avian isolates. On a positive note, the isolates maintained susceptibility to several other antibiotics, providing potential avenues for effective treatment. The detection of virulence-associated genes in a subset of isolates underscores the pathogenic potential of *P. aeruginosa* in apparently healthy quail birds. The absence of certain virulence genes also sheds light on the genetic diversity of these isolates. Finally, apparently healthy quail birds in the Mymensingh and Dhaka divisions of Bangladesh may carry multi-drug-resistant and virulent *Pseudomonas aeruginosa*. This is the first molecular detection, genetic characterization, virulence profiling of *P. aeruginosa* from quail in Bangladesh.

Acknowledgements

Funding**:** The research was funded by the project "Molecular detection, characterization, antibiogram, and on-site molecular detection of *R. anatipestifer* from ducks (*Anas platyrhynchos domesticus*) of different areas of Bangladesh" (Project No.: 2021/83/BAU), a grant from Bangladesh Agricultural University Research System (BAURES), Bangladesh Agricultural University, Mymensingh-2202, Bangladesh

Author contributions: AH: conducted the main research; MTA: equally contributed to the research work and sequence analysis and related works, BBRP: contributed to the research work and final preparation of initial draft; AR and TI: edited and prepared final draft; CSC:

risk factors analysis, CSC; AKMAR: data analysis; MSI: critically reviewed the manuscript; MPS: planned, designed and supervised the research work

Disclosure

There is no conflict of interest as declared by all the authors.

References

1. Das SC, Chowdhury SD, Khatun MA, Nishibori M, Isobe N, and Yoshimura Y. Poultry production profile and expected future projection in Bangladesh. Worlds Poult. Sci. J. 2008; 64: 99-118.

2. Redoy MR, Shuvo AA, and Al-Mamun M. A review on present status, problems and prospects of quail farming in Bangladesh. Bang. J. Anim. Sci. 2017; 46:109-20.

3. Rahman ANMA, Hoque MN, Talukder AK, and Das ZC. A survey of Japanese quail (Coturnix coturnix japonica) farming in selected areas of Bangladesh. Vet. World. 2016; 9: 940-947.

4. Ojedapo LO, and Amao SR. Sexual dimorphism on carcass characteristics of Japanese quail (Coturnix coturnix japonica) reared in derived Savanna zone of Nigeria. Int. J. Sci. Environ. Technol. 2014; 3:250-257.

5. Islam MT, Talukder AK, Rahman MA, Haider MG, Rahman ANMA. Incidence of diseases in Japanese quail (Coturnix coturnix japonica) with special reference to bacterial and viral diseases in some selected areas of Bangladesh. Asian-Australasian J. Biosci. Biotechnol. 2016; 1:410-418.

6. Dipineto L, Russo TP, Gargiulo A, Borrelli L, De Luca Bossa LM, Santaniello A, Buonocore P, Menna LF, and Fioretti A. Prevalence of enteropathogenic bacteria in

common quail (Coturnix coturnix). Avian Pathol. 2014; 43(6): 498-500.

7. Badr JM, Metwali AS, Yoseif AI, and Arafa MM. Treatment trials of *Pseudomonas aeruginosa* infection in quails. J. Vet. Med. Res. 2006; 16:46-60.

8. Martinez-Laorden A, Arraiz-Fernandez C, and Gonzalez-Fandos E. Microbiological quality and safety of fresh quail meat at the retail level. Microorganisms. 2023; 11:2213.

9. Hafez HM, Woorrak H, and Heil G. Pseudomonas infection in turkey poults and treatment trials with apramycin. Berl. Munch. Tierarztl. Wochenschr. 1987; 100:48-51.

10. El-Shorbagy MA, Mosaad AA, Hussein BM, and Abd Allah MM. Studies on *Pseudomonas aeruginosa* infection in quails. J. Egypt. Vet. Med. Assoc. 2001; 61:205-221.

11. Abd El-Baky RM, Masoud SM, Mohamed DS, Waly NG, Shafik EA, Mohareb DA, Elkady A, Elbadr MM, and Hetta HF. Prevalence and some possible mechanisms of colistin resistance among multidrug-resistant and extensively drug-resistant *Pseudomonas aeruginosa* . Infect. Drug Resist. 2020; 13:323–32.

12. Reynolds D, and Kollef M. The epidemiology and pathogenesis and treatment of *Pseudomonas aeruginosa* infections: an update. Drugs. 2021;81(18):2117–31. https://doi.org/10.1007/s40265-021-01635-6.

13. Faraji F, Mahzounieh M, Ebrahimi A, Fallah F, Teymournejad O, and Lajevardi B. Molecular detection of virulence genes in *Pseudomonas aeruginosa* isolated from children with Cystic Fibrosis and burn wounds in Iran. Microb. Pathog. 2016; 99:1–4.

14. Ma Q, Zhai Y, Schneider JC, Ramseier TM, Saier Jr MH. Protein secretion systems of *Pseudomonas aeruginosa* and P. fluorescens. Biochim. Biophys. Acta. 2003; 11:223-33. doi: 10.1016/S0005-2736(03)00059-2.

15. Filloux A, Hachani A, and Bleves. The bacterial type VI secretion machine: yet another player for protein transport across membranes. Microbiology. 2008; 154:1570-83. doi: 10.1099/mic.0.2008/016840-0.

16. McMorran B, Town L, Costelloe E, Palmer J, Engel J, Hume D, and Wainwright B. Effector ExoU from the type III secretion system is an important modulator of gene expression in lung epithelial cells in response to *Pseudomonas aeruginosa* infection. Infect. Immun. 2003; 71:6035-44. doi: 10.1128/IAI.71.10.6035-6044.2003.

17. Kazmierczak BI, and Engel JN. *Pseudomonas aeruginosa* ExoT acts in vivo as a GTPase-activating protein for RhoA, Rac1, and Cdc42. Infect. Immun. 2002; 70:2198-205. doi: 10.1128/IAI.70.4.2198-2205.2002.

18. Hossein HM, Mehdi RM, Masoumeh A, Gholamreza A, and Masoud DM. Molecular evaluation of *Pseudomonas aeruginosa* isolated from patients in burn ward, ICU, and ITU, in a number of hospitals in Kerman province. Indian J. Fundam. Appl. Life Sci. 2015; 5: 1428-1431.

19. Azimi S, Kafil HS, Baghi HB, Shokrian S, Najaf K, Asgharzadeh M, Yousefi M, Shahrivar F. and Aghazadeh M. Presence of exoY, exoS, exoU and exoT genes, antibiotic resistance and biofilm production among *Pseudomonas aeruginosa* isolates in Northwest Iran. GMS Hyg. Infect. Control. 2016; 11.

20. Rumbaugh KP, Griswold JA, and Hamood AN. The role of quorum sensing in the in vivo virulence of *Pseudomonas aeruginosa* . Microbes Infect. 2000; 2:1721–1731.

21. Islam R, Ferdous FB, Hoque MN, Asif NA, Rana ML, Siddique MP, et al. Characterization of β-lactamase and virulence genes in *Pseudomonas aeruginosa* isolated from clinical, environmental and poultry sources in Bangladesh. PLoS One [Internet]. 2024;19(4 April):1–15. Available from:

http://dx.doi.org/10.1371/journal.pone.0296542

22. Sanya DRA, Onésime D, Vizzarro G. Recent advances in therapeutic targets identification and development of treatment strategies towards *Pseudomonas aeruginosa* infections. BMC Microbiol 2023; 23: 86. https://doi.org/10.1186/s12866-023-02832-x).

23. Elbehiry A, Marzouk E, Aldubaib M, Moussa I, Abalkhail A, Ibrahem M, Hamada M, Sindi W, Alzaben F, Almuzaini AM, Algammal AM, and Rawway M. Pseudomonas species prevalence, protein analysis, and antibiotic resistance: an evolving public health challenge. AMB Express. 2022; 12(1):53. doi: 10.1186/s13568-022-01390-1.

24. Shafiq M, Zeng M, Permana B, Bilal H, Huang J, Yao F, Algammal AM, Li X, Yuan Y, Jiao X. Coexistence of blaNDM-5 and tet(X4) in international high-risk Escherichia coli clone ST648 of human origin in China. Front Microbiol. 2022; 13:1031688. doi: 10.3389/fmicb.2022.1031688.

25. Algammal AM, Eidaroos NH, Alfifi KJ, Alatawy M, Al-Harbi AI, Alanazi YF, Ghobashy MO, Khafagy AR, Esawy AM, El-Sadda SS, and Hetta HF. opr L gene sequencing, resistance patterns, virulence genes, quorum sensing and antibiotic resistance genes of XDR *Pseudomonas aeruginosa* isolated from broiler chickens. Infect. Drug Resist. 2023; 31: 853-67.

26. Algammal AM, Abo Hashem ME, Alfifi KJ, Al-Otaibi AS, Alatawy M, ElTarabili RM, Abd El-Ghany WA, Hetta HF, Hamouda AM, Elewa AA, and Azab MM. Sequence analysis, antibiogram profile, virulence and antibiotic resistance genes of XDR and MDR Gallibacterium anatis isolated from layer chickens in Egypt. Infect Drug Resist. 2022; 15: 4321-4334. doi: 10.2147/IDR.S377797.

27. Al-Kadmy IMS, Abid SA, Aziz SN, Al-Kadmy Z, Suhail A, Al-Jubori SS, Naji EN,

Alhomaidi E, Yahia R, Algammal A M, Batiha GE, and Hetta HF. The secrets of environmental *Pseudomonas aeruginosa* in slaughterhouses: Antibiogram profile, virulence, and antibiotic resistance genes. Folia Microbiol (Praha). 2023; doi: 10.1007/s12223-023-01116-1. Epub ahead of print.

28. Allcock S, Young EH, Holmes M, Gurdasani D, Dougan G, Sandhu MS, and Török ME. Antimicrobial resistance in human populations: challenges and opportunities–ERRATUM. Glob. Health Epidemiol. Genom. 2. 2017.

29. Anane AY, Apalata T, Vasaikar S, Okuthe GE, and Songca S. Prevalence and molecular analysis of multidrug-resistant Acinetobacter baumannii in the extra-hospital environment in Mthatha, South Africa. Braz. J. Infect. Dis. 2020; 23:371-80.

30. Siddique MP, Jang WJ, Lee JM, Ahn SH, Suraiya S, Kim CH, and Kong IS. groEL is a suitable genetic marker for detecting Vibrio parahaemolyticus by loop-mediated isothermal amplification assay. Lett. Appl. Microbiol, 2017; 65:106-113.

31. Spilker T, Coenye T, Vandamme P, and Lipuma JJ. PCR-based assay for differentiation of *Pseudomonas aeruginosa* from other Pseudomonas species recovered from cystic fibrosis patients. J. Clin. Microbiol. 2004; 42:2074-2079.

32. Pettit TJ, Croxton KL, Fiksel J. Ensuring supply chain resilience: Development and implementation of an assessment tool. J Bus Logist. 2013;34(1):46–76.

33. Bauer A, Kirby W, Sherris JC, and Turck M. Antibiotic susceptibility testing by a standardized single disk method. Am. J. Clin. Pathol. 1966; 45:493-496.

34. CLSI. 2019. Performance Standards for Antimicrobial Susceptibility Testing, M100. 29th ed. Clinical and Laboratory Standards Institute, Wayne, PA.

35. R Core Team. RA Language and Environment for Statistical Computing. R Foundation for Statistical Computing, Vienna, Austria. 2023. https://www.r-project.org/

36. Chongsuvivatwong V. epiDisplay: Epidemiological Data Display Package. R Package Version 3.5. R Core Team: Vienna, Austria. 2022.

37. Hall TA. BioEdit: a user-friendly biological sequence alignment editor and analysis program for Windows 95/98/NT. Nucleic Acids Symp. Ser.1999; 41:95-98.

38. Tamura K, Stecher G, Kumar S. MEGA11: molecular evolutionary genetics analysis version 11. Mol. Biol. Evol. 2021; 38:3022-3027.

39. Letunic I, and Bork P. Interactive Tree of Life (iTOL) v5: an online tool for phylogenetic tree display and annotation. Nucleic Acids Res. 2021; 49(W1): W293-296.

40. Gong Q, Ruan MD, Niu MF, Qin CL, Hou Y, and Guo JZ. Immune efficacy of DNA vaccines based on oprL and oprF genes of *Pseudomonas aeruginosa* in chickens. Poult. Sci. 2018;97: 4219-4227.

41. Bakheet AA, and Torra DE. Detection of *Pseudomonas aeruginosa* in Dead Chicken Embryo with Reference to Pathological Changes and Virulence Genes. Alex. J. Vet. Sci. 2020; 65.

42. Algammal A, Hetta HF, Mabrok M, and Behzadi P. Editorial: Emerging multidrug-resistant bacterial pathogens "superbugs": A rising public health threat. Front Microbiol. 2023; 4:1135614. doi: 10.3389/fmicb.2023.1135614.

43. Abd El-Tawab A, Awad A, Kabil A, Elhofy F, Soliman E, and Nada F. Bacteriological studies on psychrotrophic bacteria and Pseudomonas isolated from frozen fish. Benha Vet. Med. J 2019; 37:118-21.

44. Shahat HS, Mohamed H, Al-Azeem A, Mohammed W, and Nasef SA. Molecular detection of some virulence genes in *Pseudomonas aeruginosa* isolated from chicken embryos and broilers with regard to disinfectant resistance. SVU- Int. J. Vet. Sci. 2019; 2:52-70.

45. Warrier R, Skoric B, Vidmar S, Carzino R, and Ranganathan S. The role of geographical location and climate on recurrent Pseudomonas infection in young children with Cystic Fibrosis. J. Cyst. Fibros. 2019; 18:817-822.

46. Abd El-Ghany WA. *Pseudomonas aeruginosa* infection of avian origin: Zoonosis and one health implications. Vet. World. 2021; 14:2155.

47. Saad ZA, Nasef SA, Elhariri M, Elhelw R, and Ezzeldeen N. Resistance patterns associated with bacterial pathogens causing omphalitis in baby chicks. Biosci. Res. 2017; 14:845–851.

48. Badr H, Roshdy H, Abd El-Hafez AES, and Farghaly EMM. Prevalence, pathogenicity and antibiogram sensitivity of *Pseudomonas aeruginosa* isolated from diseased chickens. Assiut Vet. Med. J. 2016; 62:119-26.

49. Hafiz TA, Bin Essa EA, Alharbi SR, Alyami AS, Alkudmani ZS, Mubaraki MA, Alturki NA, and Alotaibi F. Epidemiological, microbiological, and clinical characteristics of multi-resistant *Pseudomonas aeruginosa* isolates in King Fahad Medical City, Riyadh, Saudi Arabia. Trop. Med. Infect. Dis. 2023; 8:205-205.

50. Tran NB, Truong QM, Nguyen LQ, Nguyen NM, Tran QH, Dinh TT, Hua VS, Nguyen VD, Lambert PA, and Nguyen TT. Prevalence and virulence of commensal *Pseudomonas aeruginosa* isolates from healthy individuals in Southern Vietnam (2018–2020). Biomedicines. 2023;11:54.

51. Chotirmall SH, Smith SG, Gunaratnam C, Cosgrove S, Dimitrov BD, O'Neill SJ, Harvey BJ, Greene CM, and McElvaney NG. Effect of estrogen on *Pseudomonas mucoidy* and exacerbations in cystic fibrosis. N. Engl. J. Med. 2012; 366:1978-86.

52. Moradali MF, Ghods S, and Rehm BH. *Pseudomonas aeruginosa* lifestyle: a paradigm for adaptation, survival, and persistence. Front. Cell. Infect. Microbiol. 2017; 7:39.

doi:10.3389/fcimb.2017.00039.

53. Roy-Burman A, Savel RH, Racine S, Swanson BL, Revadigar NS, Fujimoto J, Sawa T, Frank DW, and Wiener-Kronish J P. Type III protein secretion is associated with death in lower respiratory and systemic *Pseudomonas aeruginosa* infections. J. Infect. Dis. 2001;183:1767-74. doi: 10.1086/320737.

54. Lee VT, Smith RS, Tümmler B, and Lory S. Activities of *Pseudomonas aeruginosa* effectors secreted by the Type III secretion system in vitro and during infection. Infect. Immun. 2005; 73:1695-705. doi: 10.1128/IAI.73.3.1695-1705.2005.

55. Wareham DW, and Curtis MA. A genotypic and phenotypic comparison of type III secretion profiles of *Pseudomonas aeruginosa* cystic fibrosis and bacteremia isolates. Int. J. Med. Microbiol. 2007; 297:227-34.

56. Ghazaei C. Molecular Analysis of Pathogenic Genes (lasB and exoA) in *Pseudomonas aeruginosa* strains isolated from animal and human samples and determination of their resistance pattern. J. Clin. Res. Paramed Sci. 2021; 10(2): e117080.

57. Bratu S, Gupta J, and Quale J. Expression of the las and rhl quorum-sensing systems in clinical isolates of *Pseudomonas aeruginosa* does not correlate with efflux pump expression or antimicrobial resistance. J. Antimicrob. Chemother. 2006; 58:1250–3.

58. Kebede F. Pseudomonas infection in chickens. J. Vet. Med. Anim. Heal. 2010; 2:55-58.

59. Habeeb RH, Saad SN, and Al-Jubory A. A study of efficacy of disinfectants and bacterial contamination in Al-hilla teaching hospital. Med. J. Babylon. 2012; 9:890- 900.

60. Walker S, Sander J, Cline J, and Helton J. Characterization of *Pseudomonas aeruginosa* isolates associated with mortality in broiler chicks. Avian Dis. 2002; 46:1045–1050.

61. Ahmed OB. Incidence and antibiotic susceptibility pattern of *Pseudomonas aeruginosa*

isolated from inpatients in two Tertiary Hospitals. Clin. Microbiol. 2016; 5:248-52.

62. Tartor YH, and El-Naenaeey EY. RT-PCR detection of exotoxin genes expression in multi-drug resistant *Pseudomonas aeruginosa* . Cell. Mol. Biol. 2016; 62:56–62.

63. Mohammad HH. Phenotypic investigation for virulence factors of pyocine producing *Pseudomonas aeruginosa* isolated from burn wounds, Iraq. Int. J. Sci. Eng. Res. 2013; 4:2114-21.

64. Abd El Tawab AA, Maarouf AA, and Ahmed NM. Detection of virulence factors of Pseudomonas species isolated from fresh water fish by PCR. Benha Vet. Med. J. 2016; 30:199-207.

65. Sandhu R, Dahiya S, and Sayal P. Evaluation of Multiple Antibiotic Resistance (MAR) Index and Doxycycline Susceptibility of Acinetobacter Species among Inpatients. Ind. J..of Microbial Res. 2016; 3, 299-304.

66. Afunwa RA, Ezeanyinka J, Afunwa EC, Udeh AS, Oli AN, Unachukwu M. Multiple antibiotic resistant index of gram-negative bacteria from bird droppings in two commercial poultries in Enugu, Nigeria. Open J. of Med. Microbiol.. 2020 Nov 4;10(4):171-81.

Figure Click here to access/download;Figure;PA_Fig.docx

Manuscript title: First time detail Virulence and antibiogram profiling of *Pseudomonas aeruginosa* isolated from apparently healthy Japanese quail (*Coturnix japonica*) in Bangladesh

Fig 1.

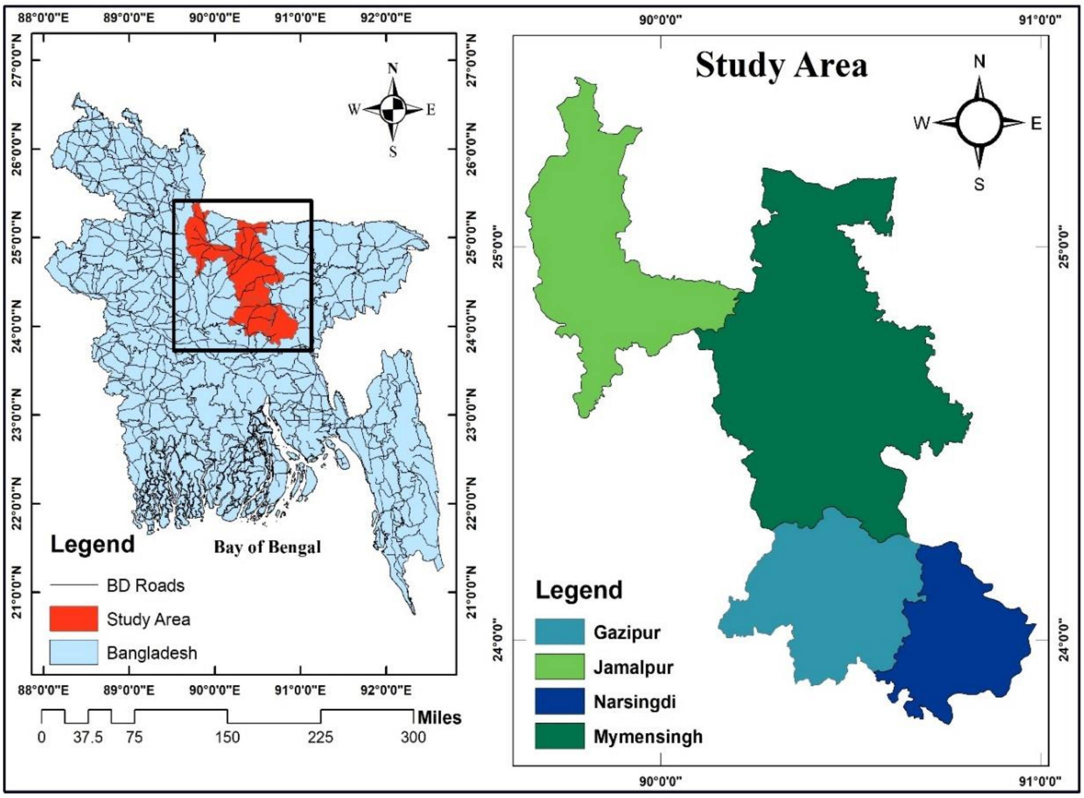

Fig 2.

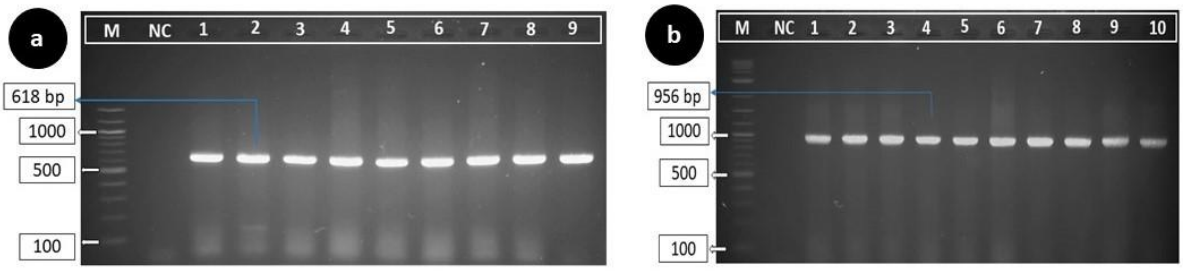
a
M
NC
1
2
3
4
5
6
7
8
9
618 bp
1000
500
100
b
M
NC
1
2
3
4
5
6
7
8
9
10
956 bp
1000
500
100

Fig 3.

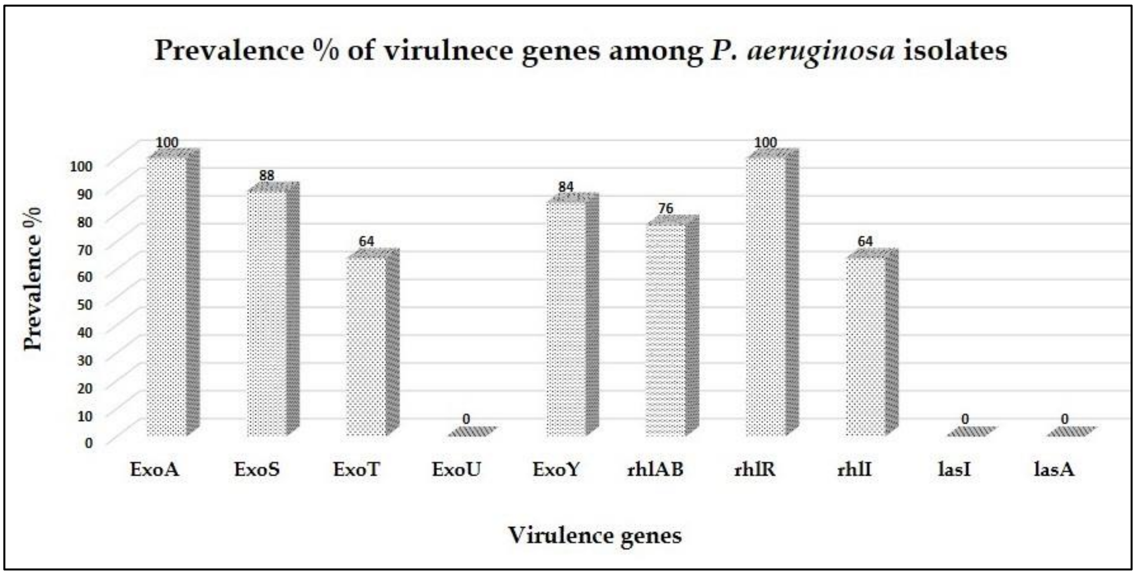

Prevalence % of virulnece genes among P. aeruginosa isolates
Prevalence %
100
90
80
70
60
50
40
30
20
10
0
100
88
64
0
84
76
100
64
0
0
ExoA
ExoS
ExoT
ExoU
ExoY
rhlAB
rhlR
rhlI
lasI
lasA
Virulence genes

Fig 4.

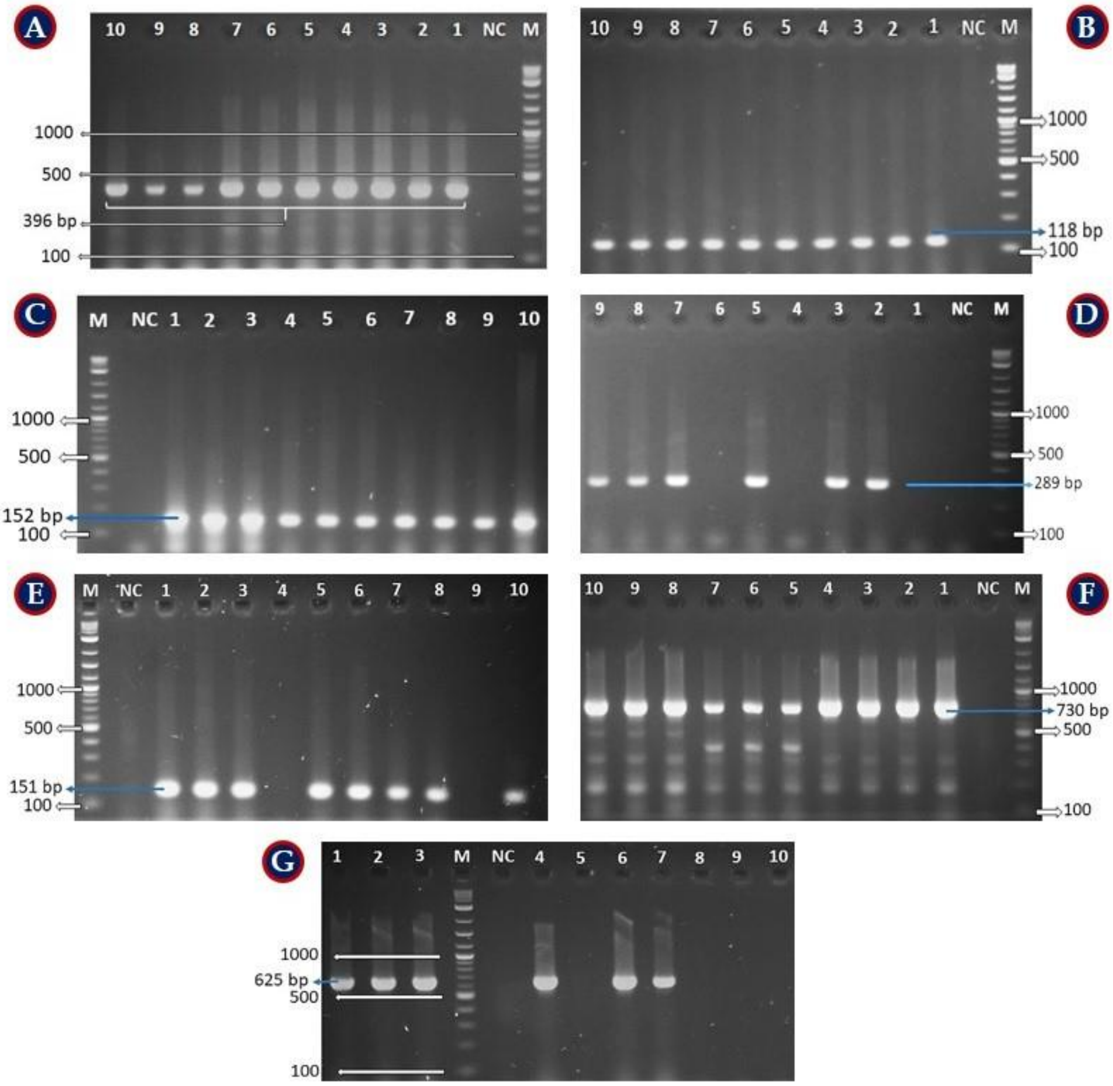
A
10 9 8 7 6 5 4 3 2 1 NC M
1000
500
396 bp
100
B
10 9 8 7 6 5 4 3 2 1 NC M
1000
500
118 bp
100
C
M NC 1 2 3 4 5 6 7 8 9 10
1000
500
152 bp
100
D
9 8 7 6 5 4 3 2 1 NC M
1000
500
289 bp
100
E
M NC 1 2 3 4 5 6 7 8 9 10
1000
500
151 bp
100
F
10 9 8 7 6 5 4 3 2 1 NC M
1000
730 bp
500
100
G
1 2 3 M NC 4 5 6 7 8 9 10
1000
625 bp
500
100

Fig 5.

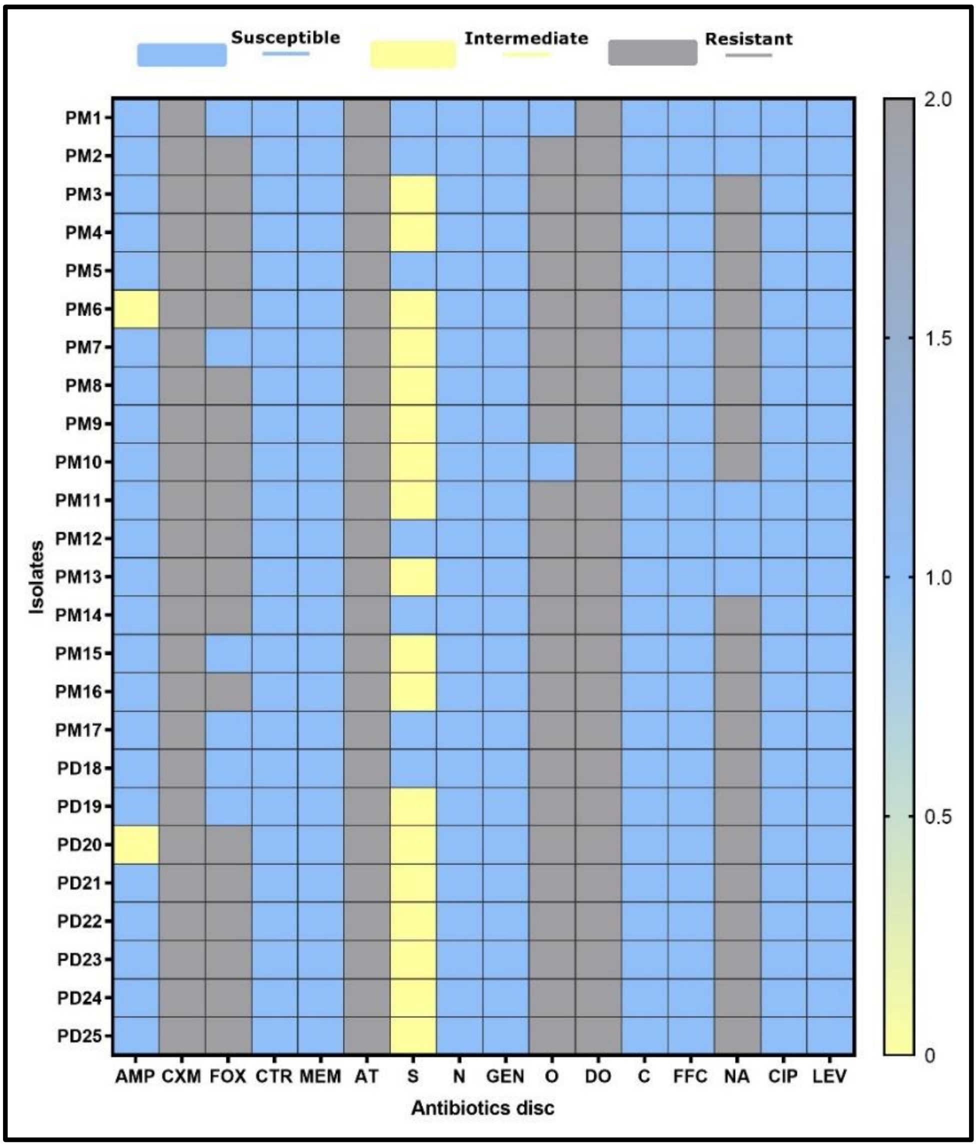
Susceptible
Intermediate
Resistant
Isolates
PM1
PM2
PM3
PM4
PM5
PM6
PM7
PM8
PM9
PM10
PM11
PM12
PM13
PM14
PM15
PM16
PM17
PD18
PD19
PD20
PD21
PD22
PD23
PD24
PD25
AMP CXM FOX CTR MEM AT S N GEN O DO C FFC NA CIP LEV
Antibiotics disc
2.0
1.5
1.0
0.5
0

Fig 6.

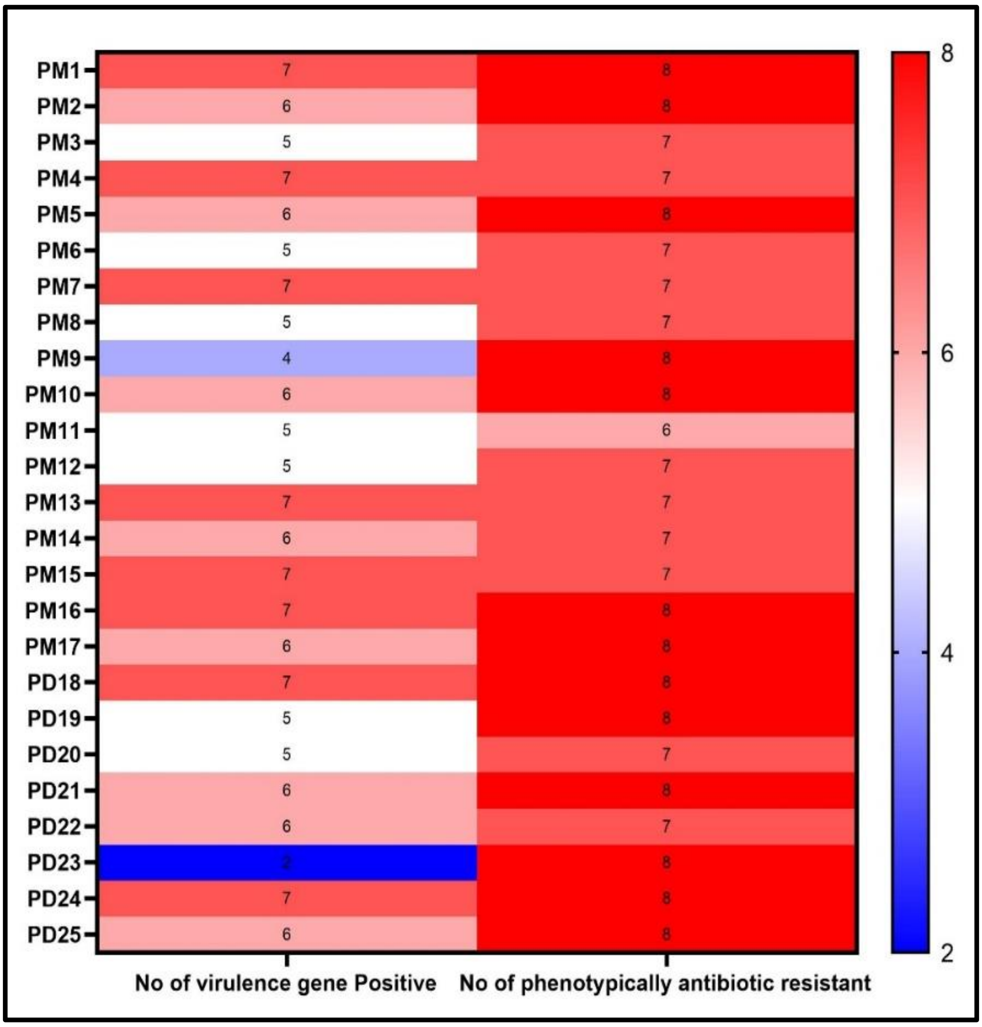
PM1
PM2
PM3
PM4
PM5
PM6
PM7
PM8
PM9
PM10
PM11
PM12
PM13
PM14
PM15
PM16
PM17
PD18
PD19
PD20
PD21
PD22
PD23
PD24
PD25
No of virulence gene Positive
No of phenotypically antibiotic resistant
8
6
4
2

Fig 7.

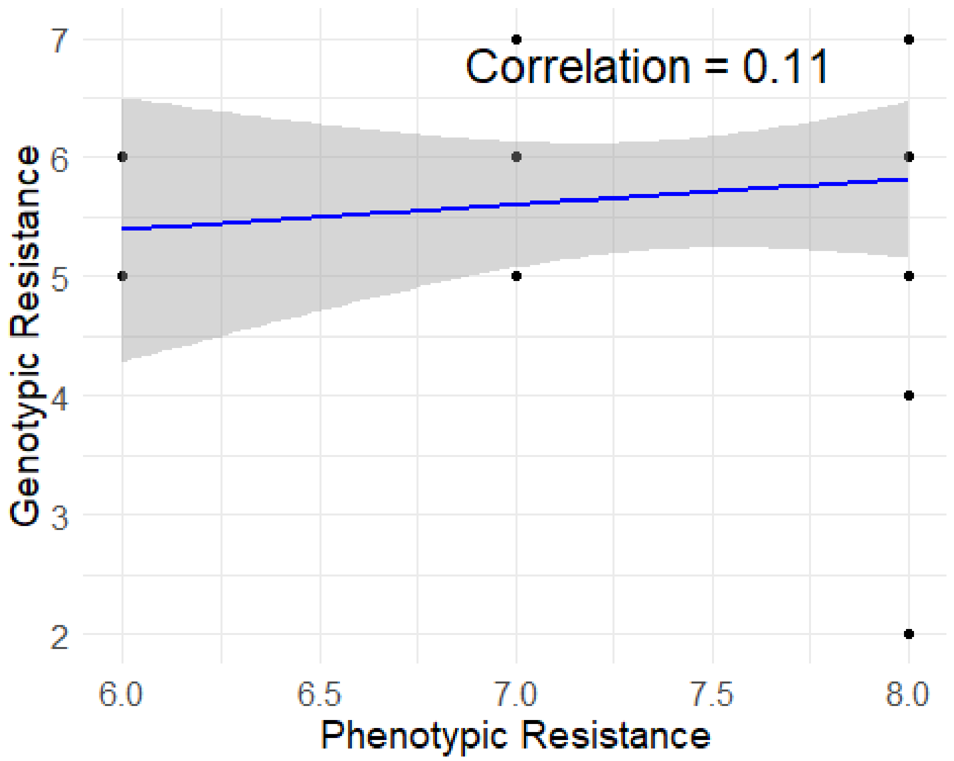

Correlation = 0.11
Genotypic Resistance
Phenotypic Resistance
7
6
5
4
3
2
6.0
6.5
7.0
7.5
8.0

Fig 8.

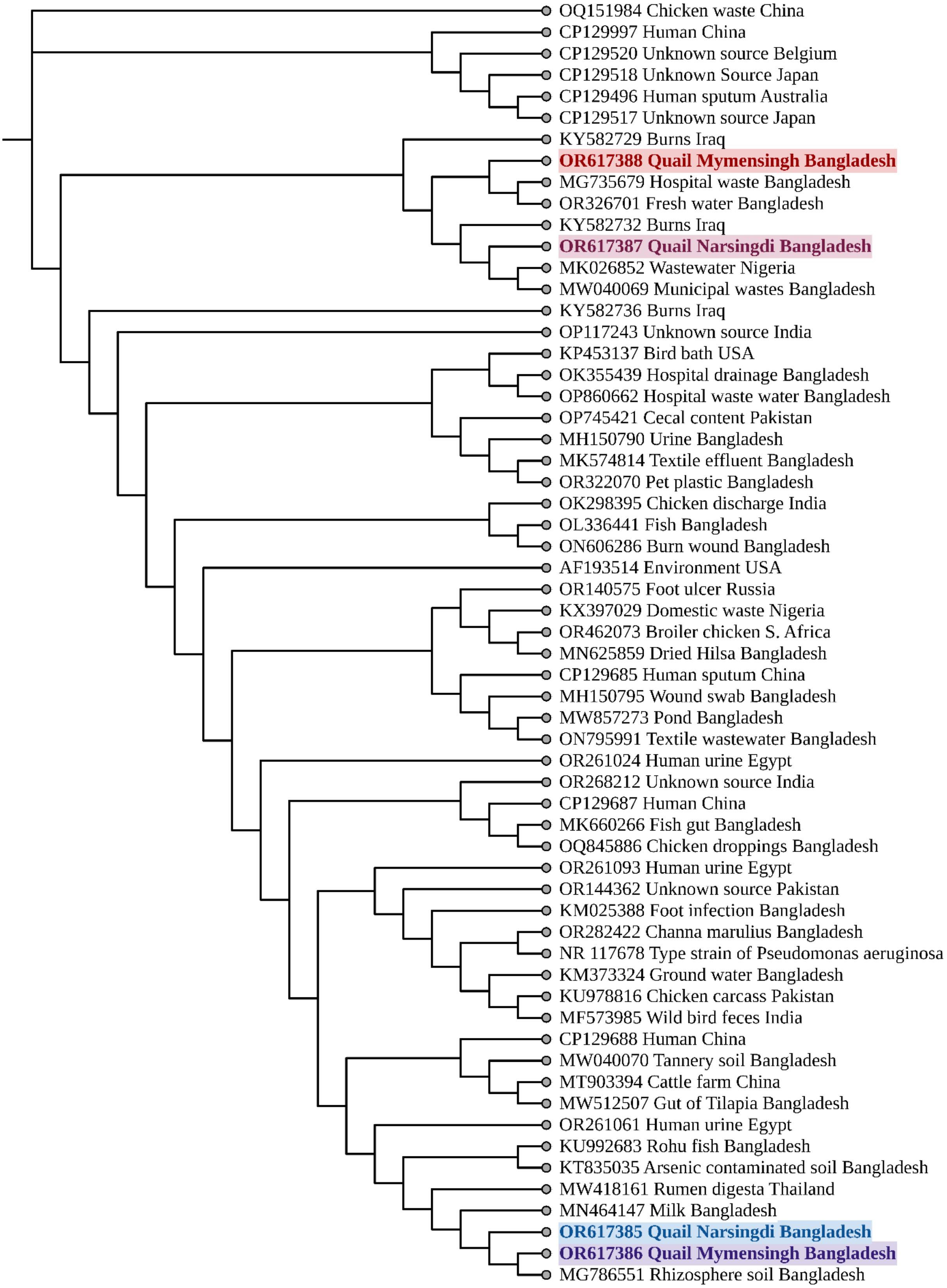
OQ151984 Chicken waste China
CP129997 Human China
CP129520 Unknown source Belgium
CP129518 Unknown Source Japan
CP129496 Human sputum Australia
CP129517 Unknown source Japan
KY582729 Burns Iraq
OR617388 Quail Mymensingh Bangladesh
MG735679 Hospital waste Bangladesh
OR326701 Fresh water Bangladesh
KY582732 Burns Iraq
OR617387 Quail Narsingdi Bangladesh
MK026852 Wastewater Nigeria
MW040069 Municipal wastes Bangladesh
KY582736 Burns Iraq
OP117243 Unknown source India
KP453137 Bird bath USA
OK355439 Hospital drainage Bangladesh
OP860662 Hospital waste water Bangladesh
OP745421 Cecal content Pakistan
MH150790 Urine Bangladesh
MK574814 Textile effluent Bangladesh
OR322070 Pet plastic Bangladesh
OK298395 Chicken discharge India
OL336441 Fish Bangladesh
ON606286 Burn wound Bangladesh
AF193514 Environment USA
OR140575 Foot ulcer Russia
KX397029 Domestic waste Nigeria
OR462073 Broiler chicken S. Africa
MN625859 Dried Hilsa Bangladesh
CP129685 Human sputum China
MH150795 Wound swab Bangladesh
MW857273 Pond Bangladesh
ON795991 Textile wastewater Bangladesh
OR261024 Human urine Egypt
OR268212 Unknown source India
CP129687 Human China
MK660266 Fish gut Bangladesh
OQ845886 Chicken droppings Bangladesh
OR261093 Human urine Egypt
OR144362 Unknown source Pakistan
KM025388 Foot infection Bangladesh
OR282422 Channa marulius Bangladesh
NR 117678 Type strain of Pseudomonas aeruginosa
KM373324 Ground water Bangladesh
KU978816 Chicken carcass Pakistan
MF573985 Wild bird feces India
CP129688 Human China
MW040070 Tannery soil Bangladesh
MT903394 Cattle farm China
MW512507 Gut of Tilapia Bangladesh
OR261061 Human urine Egypt
KU992683 Rohu fish Bangladesh
KT835035 Arsenic contaminated soil Bangladesh
MW418161 Rumen digesta Thailand
MN464147 Milk Bangladesh
OR617385 Quail Narsingdi Bangladesh
OR617386 Quail Mymensingh Bangladesh
MG786551 Rhizosphere soil Bangladesh
